# Evaluation of three industrial *Escherichia coli* strains in fed-batch cultivations during high-level SOD protein production

**DOI:** 10.1186/1475-2859-12-58

**Published:** 2013-06-11

**Authors:** Karoline Marisch, Karl Bayer, Monika Cserjan-Puschmann, Markus Luchner, Gerald Striedner

**Affiliations:** 1 Department of Biotechnology, University of Natural Resources and Life Sciences, Muthgasse 18, 1190, Vienna, Austria; 2 Austrian Centre of Industrial Biotechnology, Muthgasse 11, 1190, Vienna, Austria

**Keywords:** *E. coli*, Fed-batch, SOD, Recombinant protein production, Bioreactor cultivation, Strain characterization

## Abstract

**Background:**

In the biopharmaceutical industry, *Escherichia coli* (*E. coli*) strains are among the most frequently used bacterial hosts for producing recombinant proteins because they allow a simple process set-up and they are Food and Drug Administration (FDA)-approved for human applications. Widespread use of *E. coli* in biotechnology has led to the development of many different strains, and selecting an ideal host to produce a specific protein of interest is an important step in developing a production process. *E. coli* B and K–12 strains are frequently employed in large-scale production processes, and therefore are of particular interest. We previously evaluated the individual cultivation characteristics of *E. coli* BL21 and the K–12 hosts RV308 and HMS174. To our knowledge, there has not yet been a detailed comparison of the individual performances of these production strains in terms of recombinant protein production and system stability. The present study directly compared the T7-based expression hosts *E. coli* BL21(DE3), RV308(DE3), and HMS174(DE3), focusing on evaluating the specific attributes of these strains in relation to high-level protein production of the model protein recombinant human superoxide dismutase (SOD). The experimental setup was an exponential carbon-limited fed-batch cultivation with minimal media and single-pulse induction.

**Results:**

The host strain BL21(DE3) produced the highest amounts of specific protein, followed by HMS174(DE3) and RV308(DE3). The expression system HMS174(DE3) exhibited system stability by retaining the expression vector over the entire process time; however, it entirely stopped growing shortly after induction. In contrast, BL21(DE3) and RV308(DE3) encountered plasmid loss but maintained growth. RV308(DE3) exhibited the lowest ppGpp concentration, which is correlated with the metabolic stress level and lowest degradation of soluble protein fraction compared to both other strains.

**Conclusions:**

Overall, this study provides novel data regarding the individual strain properties and production capabilities, which will enable targeted strain selection for producing a specific protein of interest. This information can be used to accelerate future process design and implementation.

## Introduction

The best applicable expression host for an individual production process is typically determined based on various characteristics and attributes of the specific recombinant protein, like glycosylation pattern or post-translational modifications. *Escherichia coli* (*E. coli*) is by far the most widely used host organism for biopharmaceutical production of simple non-modified heterologous recombinant proteins with a low number of disulfide bonds. *E. coli* expression strains are favored due to their ability to quickly reach high cell densities in inexpensive media and their Food and Drug Administration (FDA)-approved status for human applications [1]. Additionally, their well-characterized genetics and publicly available genome sequences [2-4], and the availability of a large number of cloning vectors and various mutant strains ensure that *E. coli* remains a valuable host for recombinant protein production [5]. However, some drawbacks must be considered when selecting an *E. coli* expression system, including the limited ability to build disulfide bonds, the lack of an efficient secretion system due to the complexity of the two cell membranes, and the inability to perform posttranslational modifications that occur with eukaryotic proteins [6,7].

Selection of an appropriate promoter is also a major step in process design. For high-level recombinant protein synthesis in *E. coli,* the promoter must be strong and inducible, and should exhibit a minimal level of basal transcriptional activity [7]. The pET system (plasmid for expression by T7 RNA polymerase) applied in the present study meets most of these requirements [8,9]; however, the resulting strong and high-level protein expression can overburden the metabolic capacities of the production host and lead to growth inhibition or even cessation after a short period of time [10,11]. Therefore, the actual production phase during a process is very limited. Strong host/vector interactions also contribute to the stress level of the host, resulting in decreased recombinant protein yields far below the theoretical maximum. High-level protein production can also lead to modifications in catabolism and anabolism, including adjustments of the energy generating system, the protein producing system, and cellular fluxes [11,12]. Overall, the cellular reactions caused by recombinant protein production are similar to the heat shock and stringent responses of *E. coli *[12]. Altogether, these unfavorable conditions and the limitation of cellular resources caused by high production rates often result in accumulation of damaged and un- or mis-folded proteins in the host cell [13]. Inclusion bodies (IB) sometimes form, depending on the specific folding behavior of the protein of interest; even small changes in the primary structure of a protein can influence its solubility [14].

Furthermore, in plasmid-based expression systems, the cellular energy required for plasmid maintenance and replication may influence the process outcome. In some cases the applied systems encounter problems with plasmid stability, leading to plasmid-free cell populations. To avoid plasmid-maintenance and stability problems, genome-based expression systems that integrate the target gene into the chromosome have been established for *E. coli *[15]; however, plasmid-based systems are still favored in industry because the cloning procedures are simpler and faster.

The present work focused on evaluating the prominent and very strong T7 expression system (commercialized by Novagen), within the most popular *E. coli* hosts, using an industrial-like process set-up. Previous comparisons of different strains have investigated biomass yield, growth behavior, or mRNA expression profiles [16-19]. For recombinant processes the impact of recombinant protein production onto the host metabolism is of also of great interest, as the host-vector interactions during such harsh conditions are often complex and unpredictable. Here we transformed the B strain BL21(DE3) and the two K–12 strains HMS174(DE3) and RV308(DE3) with the pET30a plasmid (Novagen; Germany) encoding the gene for human superoxide dismutase (SOD, EC 1.15.1.1) [20], and we evaluated their performances in fully induced fed-batch cultivations. The two K–12 hosts, RV308 a derivative of MG1655 and HMS174 of W3110, were compared to the well-known BL21 strain. All three are industrially relevant strains frequently used in large scale production processes [21]. The model protein human superoxide dismutase used in this study can be expressed in soluble form in the cytoplasm of *E. coli*[20]. However, strong expression systems and high amounts of inducer (IPTG) can lead to the accumulation of both soluble and insoluble SOD (inclusion bodies; IBs). Therefore, this protein was selected as model protein and the distribution of the soluble versus the insoluble protein form was monitored and used as quality criteria. We characterized the studied strains in terms of growth behavior, productivity, product quality (solubility), and overall system stability. The experiments were designed to provide valuable information about *E. coli* performance in industrial-like processes, which can be used in future process set-up and design.

## Results

### Experimental set-up of cultivations

The key objective of this work was to evaluate the impact of T7-based protein expression in three different *E. coli* hosts in terms of growth, productivity, product quality, and system stability. The two K–12 strains RV308(DE3) (genotype: *lacI*q-, su-, Δ*lacX74*, *gal*, IS II::OP308, *strA,* (DE3)) and HMS174(DE3) (genotype: F^-^, *recA1*, *hsdR*(r_K12_^-^ m_K12_^+^) (Rif^R^) (DE3)) and the B strain BL21(DE3) (genotype: F^-^, *dcm*, *ompT*, *hsdS*(r_B_- m_B_-), *gal,* (DE3)) were cultivated during the expression of plasmid-encoded (pET30a) recombinant SOD in glucose-limited exponential fed-batch cultivations at a growth rate of 0.1 h^-1^. The following expression systems were used in this study: *E. coli* BL21(DE3)(pET30aSOD), *E. coli* HMS174(DE3)(pET30aSOD), and *E. coli* RV308(DE3)(pET30aSOD). Hereafter, these systems are referred to as BL21, HMS174, and RV308, respectively. To trigger very high recombinant gene expression levels, we applied single-pulse full induction using isopropyl β-D-1-thiogalactopyranoside (IPTG) at one doubling past the feed start. In contrast to common industrial processes, we performed an early induction of recombinant protein synthesis to allow an elongated observation time of the hosts during the production phase. Therefore, an experimental design was chosen to monitor cellular host reactions on strong recombinant protein expression under fed-batch conditions over a period of three generations (doubling times, 21 hours) in detail. All cultivations were conducted in triplicate, and samples were drawn at hourly intervals for comprehensive analysis via off-line methods, including determination of the optical density (OD600), cell number, cell viability, cell size by fluorescence-activated cell sorting (FACS), and system stability (Koch-plating). Sample aliquots were also stored for subsequent analyses of product yield, distribution between the soluble and insoluble protein fraction (product quality), plasmid copy number (PCN), guanosine tetraphosphate (ppGpp) level, and determination of metabolites (acetate, formate, pyruvate, and glucose) in the cultivation broth supernatant. The analysis of metabolites combined with off-gas analysis of O2 and CO2 also enabled calculation of mass-balances. All of these analyses were used to characterize the cellular host reactions triggered by recombinant gene expression, and the SOD production capabilities of the three strains.

### Cell growth

The actual glucose yield coefficient (Yx/s) of each strain was determined during the non-induced phase of the process. Y_X/S_ was calculated with 0.40 g cell dry mass (CDM) per g glucose for BL21 and RV308, and 0.34 g CDM per g glucose for HMS174, theoretically yielding 441.5 g CDM each for BL21 and RV308 and 372.3 g CDM for HMS174 from the total of ~1100 g glucose provided during the process. This theoretical biomass yield was compared to the measured CDM of each host.

#### Analysis of biomass formation and growth rate

**Figure 1:**
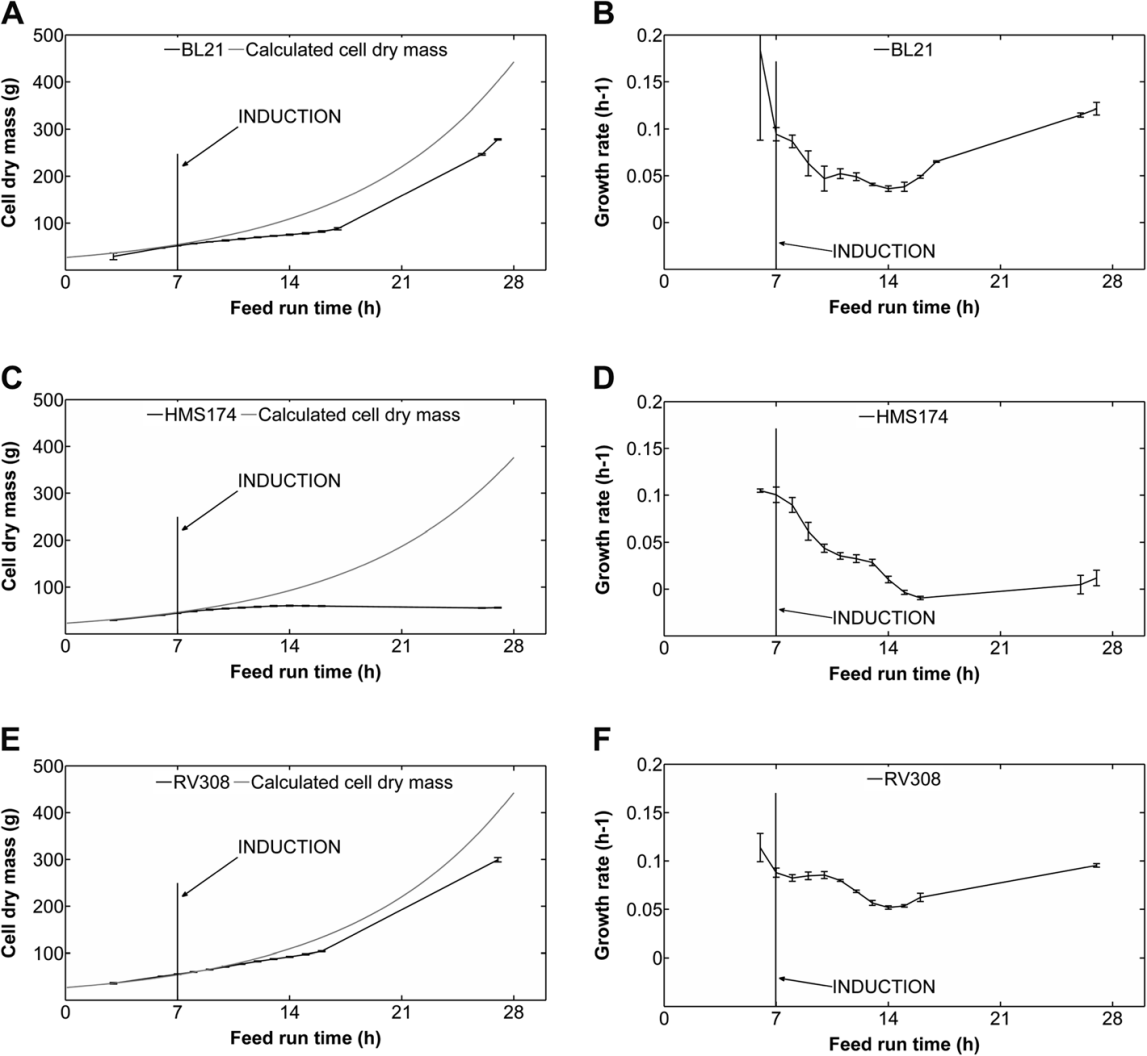
**Cell dry mass and growth rates.** Measured cell dry mass (CDM) (means and standard error of the mean) and the theoretically achievable CDM for (**A**) BL21, (**C**) HMS174, and (**E**) RV308. The theoretical CDM was calculated with constant yield coefficients of 0.34 g/g for HMS174 and 0.40 g/g for BL21 and RV308. Measured growth rates of the hosts during the fed-batch phase (means ± standard error of the mean) are shown for (**B**) BL21, (**D**) HMS174, and (**F**) RV308. Recombinant protein synthesis was induced at feed-hour seven.

Analyses of CDM during the cultivations showed different biomass courses of the three hosts, as well as deviations from the calculated specific CDM after induction of recombinant protein synthesis (Figure 1A, C and E). Each strain exhibited growth rate reduction shortly after induction (Figure 1B, D and F). BL21 and HMS174 exhibited sudden decreases in their growth rates after induction at feed-hour seven, while RV308 maintained post-induction cellular growth for four hours longer. HMS174 did not resume cellular growth; the growth rate dropped to zero and remained at zero until the end of the cultivations (Figure 1D). In contrast, growth of BL21 and RV308 resumed at a later stage of the process (feed-hour 14; Figure 1B and F). At the end of the process, BL21 and RV308 had achieved similar CDM (317 ± 7 and 317 ± 2 g CDM, respectively), while that of HMS174 was lower (57 ± 1 g CDM). These values were lower than the calculated total CDM by 28% for both BL21 and RV308, and 85% for HMS174.

#### Carbon (C) balances

To elucidate the differences in growth and carbon utilization of the three strains, C balances were calculated (Table 1). The elemental composition of *E. coli* biomass (CH_1.77_O_0.49_ N_0.24_) was derived from literature and applied for all three hosts [22,23]. The uptake of carbon provided through glucose was compared with the biomass and metabolites produced during the process. In a completely defined system, all supplied carbon can be found in components produced during the process. In addition to the carbon balance of the entire process (feed-hours 0–28), separate C balances were calculated for three phases of the process to examine the different growth kinetics of the three hosts. Phase I was the non-induced part of the process (feed-hours 0–7), phase II was the production period (feed-hours 7–16), and phase III was the final part of the process (feed-hours 16–28).

**Table 1:** Carbon balance of HMS174, RV308, and BL21 cultivations for the entire process and the separate process phases (non-induced phase, production phase, and end phase)

**HMS174**		**Experiment (0 to 28 h)**		**Phase I (0 to 7 h)**		**Phase II (7 to 16 h)**		**Phase III (16 to 28 h)**	
		**g**	**%**	**g**	**%**	**g**	**%**	**g**	**%**
*Glucose provided*		*1095.0*	***50.6***	*135.3*	***100.0***	*197.9*	***91.1***	*761. 5*	***31.3***
***Glucose consumed***		*553.8* ± 31.83		*135.3* ± 022		*180.4* ± 0.93		*238.1* ± 31.83	
C in consumed glucose	C_6_H_12_O_6_	201.4 ± 11.58		49.2 ± 0.08		65.6 ± 0.34		86.6 ± 11.32	
C in biomass	CH_1.77_O_0.49_ N_0.24_	28.5 ± 0.23	14.2	21.4 ± 0.20	43.4	7.2 ± 0.05	10.9	non-detectable	
C in acetate	CH_3_COO^-^	8.9 ± 0.64	4.4	non-detectable		non-detectable		8.9 ± 0.68	10.2
C in formate	HCOO^-^	non-detectable		non-detectable		non-detectable		non-detectable	
C in pyruvate	C_3_H_3_O_3_	2.4 ± 0.10	1.2	non-detectable		0.1 ± 0.00	0.2	2.3 ± 0.09	2.7
C in carbon dioxide	CO_2_	86.6 ± 2.77	43.0	23.1 ± 1.31	46.9	34.7 ± 1.88	52.9	28.9 ± 0.53	33.4
**Sum of C detected**		126.5	62.8	44.4	90.3	41.9	64.0	40.1	46.3
**C not detected**		74.8	**37.2**	4.8	**9.7**	23.6	**36.0**	46.5	**53.7**
**RV308**		**Experiment (0 to 28 h)**		**Phase I (0 to 7 h)**		**Phase II (7 to 16 h)**		**Phase III (16 to 28 h)**	
		**g**	**%**	**g**	**%**	**g**	**%**	**g**	**%**
*Glucose provided*		*1103.6*	***100.0***	*135.3*	***100.0***	*197.1*	***100.0***	*771.3*	***100.0***
***Glucose consumed***		*1103.6* ± 0.00		*135.3* ± 0.00		*197.1* ± 0.00		*771.3* ± 0.00	
C in consumed glucose	C_6_H_12_O_6_	401.3 ± 0.00		49.2		71.7		280.5	
C in biomass	CH_1.77_O_0.49_ N_0.24_	152.5 ± 0.46	38.0	26.7	54.3	23.2	32.3	102.7	36.6
C in acetate	CH_3_COO^-^	0.3 ± 0.04	0.1	non-detectable		0.1	0.1	0.2	0.1
C in formate	HCOO^-^	0.4 ± 0.07	0.1	non-detectable		0.1	0.1	0.3	0.1
C in pyruvate	C_3_H_3_O_3_	non-detectable		non-detectable		non-detectable		non-detectable	
C in carbon dioxide	CO_2_	190.1 ± 3.51	47.4	19.6 ± 0.21	39.9	40.5 ± 1.59	56.6	130.0 ± 2.24	46.3
**Sum of C detected**		343.3	85.5	46.3	94.1	63.8	89.0	233.2	83.1
**C not detected**		58.0	**14.5**	2.9	**5.9**	7.9	**11.0**	47.3	**16.9**
**BL21**		**Experiment (0 to 28 h)**		**Phase I (0 to 7 h)**		**Phase II (7 to 16 h)**		**Phase III (16 to 28 h)**	
		**g**	**%**	**g**	**%**	**g**	**%**	**g**	**%**
*Glucose provided*		*1103.8*	***99.8***	*135.6*	***99.8***	*197.9*	***99.6***	*770.6*	***99.9***
***Glucose consumed***		*1101.6* ± 0.01		*135.3* ± 0.03		*196.8* ± 0.15		*769.6* ± 0.11	
C in consumed glucose	C_6_H_12_O_6_	400.7 ± 0.00		49.2 ± 0.01		71.6 ± 0.05		279.8 ± 0.04	
C in biomass	CH_1.77_O_0.49_ N_0.24_	152.2 ± 2.02	38.0	24.9 ± 0.03	50.4	14.6 ± 0.46	20.4	112.7 ± 1.56	40.3
C in acetate	CH_3_COO^-^	0.6 ± 0.10	0.1	non-detectable		0.3 ± 0.04	0.4	0.3 ± 0.10	0.1
C in formate	HCOO^-^	0.3 ± 0.09	0.0	0.1 ± 0.03	0.1	0.1 ± 0.04	0.1	0.2 ± 0.07	0.1
C in pyruvate	C_3_H_3_O_3_	0.1 ± 0.10	0.0	non-detectable		non-detectable		0.1 ± 0.05	0.0
C in carbon dioxide	CO_2_	216.5 ± 1.16	54.0	25.5 ± 0.54	51.8	48.2 ± 0.50	67.3	142.8 ± 1.26	51.0
**Sum of C detected**		369.4	92.1	50.5	102.6	63.1	88.1	256.1	91.5
**C not detected**		31.3	**7.9**	-1.3	**-2.6**	8.5	**11.9**	23.8	**8.5**

During phase I, similar results were observed for the three hosts (Table 1). The carbon provided was mostly converted to biomass and carbon dioxide, but a small portion of glucose (below 10%) was not detected in any specific compounds measured. In phase II (feed-hours 7–16), biomass formation of HMS174 was significantly reduced; only 11% of C was drained to biomass, 53% to CO_2_, and about 36% of C was not assignable to measured metabolites. For the other two strains, the C balances did not show large deviations between carbon provided and detected; the non-assignable carbon fractions (~12%) were only slightly greater after induction compared to during the non-induced phase. During phase III (feed-hours 16–28) the situation was even worse for HMS174; only 31% of the glucose provided during the feed-phase was consumed, almost 54% of this carbon could not be assigned to measured metabolites, and no biomass was produced at all. Therefore, high concentrations of glucose were present in the fermentation broth of HMS174. In contrast, no residual glucose was detected in the supernatant of RV308 and BL21 cultivations; thereby the strains were growing according to the provided feed-regime.

### Recombinant superoxide dismutase protein production

Because of the different growth behaviors of the three strains, protein production was only monitored for the first nine hours past induction to enable equitable comparisons of the specific production capabilities of the three hosts. Additionally, an end-point sample of each strain was quantified to further characterize the processes, the protein expression properties, and the proteolytic activity in each system.

#### Protein yield and formation kinetics

**Figure 2:**
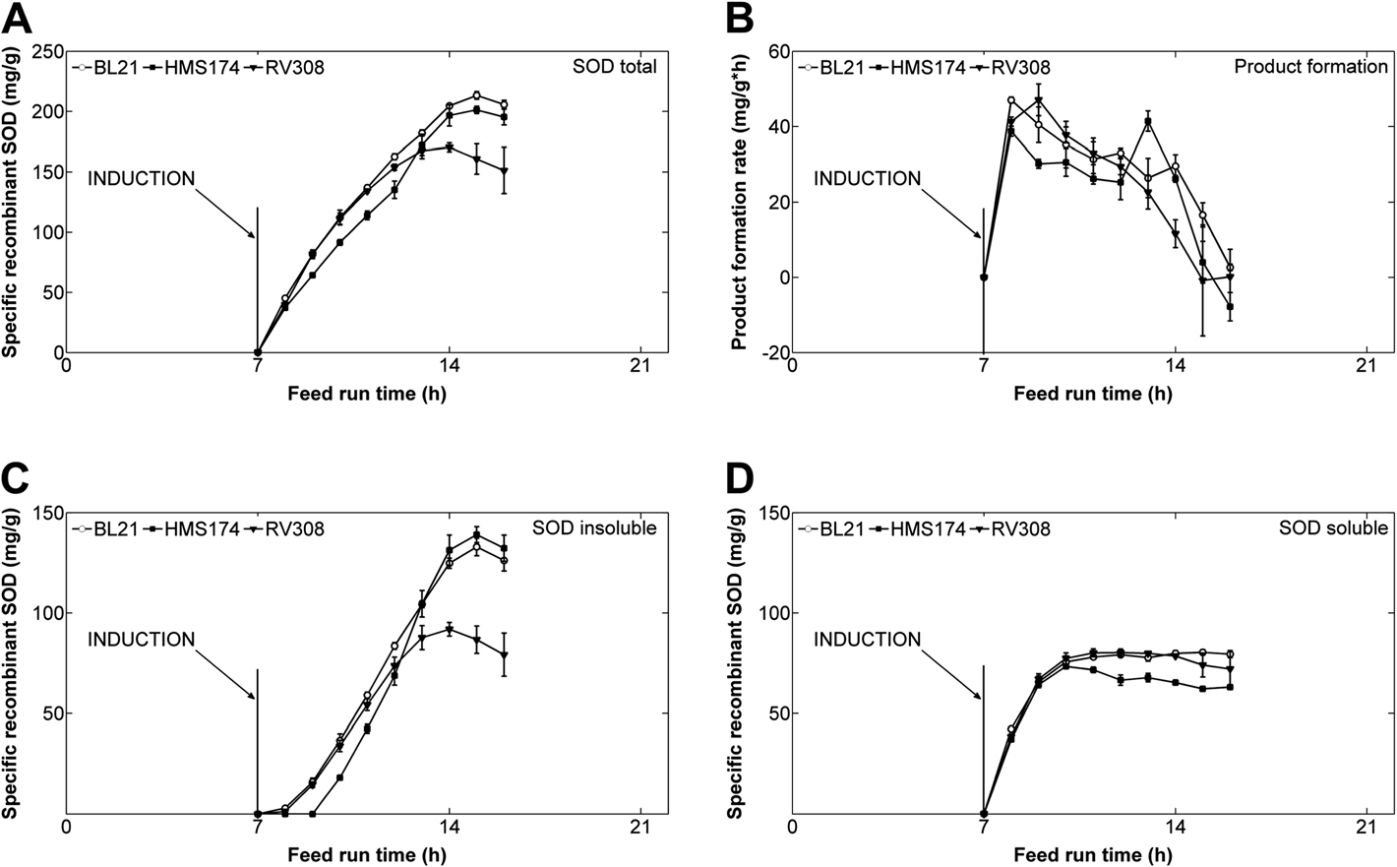
**Recombinant SOD protein synthesis.** (**A**) Total (IB and soluble) specific recombinant protein yields, (**B**) specific product formation rate (qP), (**C**) insoluble SOD fraction, and (**D**) soluble fraction of SOD for the three strains (means ± standard error of the mean). Induction of recombinant protein synthesis was performed at feed-hour 7.

The highest specific product (SOD) contents in BL21 and HMS174 were detected at eight hours post-induction (213 ± 3 and 201 ± 3 mg/g, respectively) (Figure 2A). For RV308, the highest specific amount (170 ± 3 mg/g) was measured at seven hours post-induction. At nine hours after induction, the specific product yields started to decline for all hosts. The specific recombinant protein formation rates (qP) behaved similarly for all strains, rising to maximum values of ~40 mg/g/h at two hours post-induction, and decreasing to zero within one doubling time (7 h) of induction (Figure 2B).

#### Distribution of soluble and insoluble protein

In terms of product quality, all three hosts were comparable and produced about 80 mg/g CDM of soluble SOD (Figure 2D). At feed-hour 11 (four hours induced) the soluble protein fraction of the HMS174 cultivations started to decline; whereby the protein amount of both other hosts stayed about constant during the monitored production phase.

The lowest number of inclusion bodies was detected during cultivations of RV308, with a maximum of 92 ± 3 mg/g at seven hours post-induction (feed-hour 14) (Figure 2C). BL21 and RV308 showed similar insoluble product formation up to feed-hour 11; thereafter a reduction of the specific inclusion body production rates of RV308 was measured. HMS174 and BL21 were similar in terms of overall inclusion body formation, producing 133 ± 4 and 139 ± 4 mg/g, respectively, although HMS174 showed no inclusion body formation for up to three hours post-induction (Figure 2C). This earlier start of inclusion body formation of BL21 and RV308 was reflected in higher qP levels shortly after induction (Figure 2B).

The corresponding percentages of soluble SOD were approximately 50% for RV308, and 40% each for BL21 and HMS174. Altogether, a decrease of the insoluble fraction was detected for all three hosts about seven hours post induction (feed-hour 14) for the K–12 strain RV308 and eight hours post induction (feed-hour 15) during the cultivations of the BL21 and HMS174 host (Figure 2C).

To generally characterize the processes and the hosts’ potential protein degrading and proteolytic activities, the soluble SOD fractions at the process end (feed-hour 28) were quantified and compared to those at feed-hour 16. The results revealed strong declines of specific SOD productions, with only 46.6 mg/g for HMS174, 26.2 mg/g for RV308, and 7.8 mg/g for BL21 at the process end, compared to approximately 80 mg/g soluble protein at feed-hour 16. ELISA results of the total protein amounts of soluble SOD at the end (feed-hour 28) showed reductions of 62% in BL21 (6.53 g compared to 2.53 g SOD) and 30% in HMS174 (3.74 g compared to 2.66 g), but an increase of 11% in RV308 (7.47 g compared to 8.25 g).

### Characterization of host cell response to recombinant protein expression

In this study the differences between the three hosts in terms of the cells’ responses to recombinant gene expression were investigated. We analyzed plasmid copy number (PCN), plasmid stability, cell viability, and cellular stress level (ppGpp level) [24,25] for each strain, to enable an in-depth evaluation and comparison.

#### Plasmid copy number and plasmid retention

**Figure 3:**
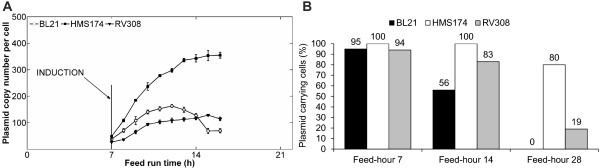
**Effects of induction on plasmid-bearing cells.** (**A**) Plasmid copy number per cell during the production phase (means ± standard error of the mean) and (**B**) percentage of plasmid-bearing cells before induction (feed-hour 7), after one generation of induction (feed-hour 14), and after three generations induced (feed-hour 28; end of process) determined by Koch-plating.

Before induction, the PCN were comparable among the strains. After induction, an increase of the PCN from 40 to nearly 400 plasmids per cell was detected within eight hours in HMS174. Both other hosts exhibited lesser influences of induction on plasmid replication, with a maximal PCN of ~160 measured for BL21 and ~130 for RV308 (Figure 3A). The percentage of plasmid-bearing cells was determined by Koch-plating. RV308 and BL21 exhibited strong decreases of plasmid-carrying cells over time (Figure 3B). In HMS174, 80% of the cells were plasmid bearing at the end of the process, while only 20% of plasmid-carrying cells remained in RV308, and none in BL21 (Figure 3B).

#### Cell viability and cell number

**Figure 4:**
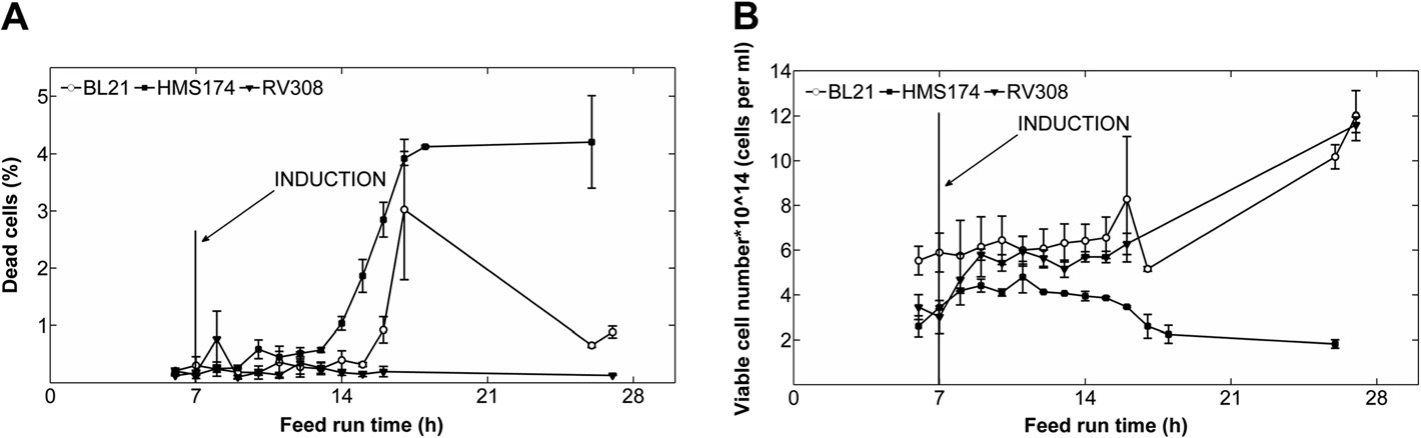
**Flow cytometric measurements of dead and viable cells.** Flow cytometric measurements of (**A**) dead cells and (**B**) viable cells during the course of the cultivations (means ± standard error of the mean). Induction of recombinant protein synthesis was performed with IPTG at feed-hour seven.

Flow cytometry revealed only very low percentages of dead cells for all hosts. For RV308, the content of dead cells was always below 1% (Figure 4A). In HMS174 and BL21, up to 3% dead cells were identified during the induced phase (feed-hours 7–16). Up to 5% dead cells were detected at the process end in HMS174 cultivations (Figure 4A), while in BL21, the number of dead cells decreased again during the course of cultivation. Flow cytometric measurements also demonstrated lower cell numbers for HMS174 compared to the other two hosts, especially at the end of the process (Figure 4B).

#### Quantification of metabolic stress

**Figure 5:**
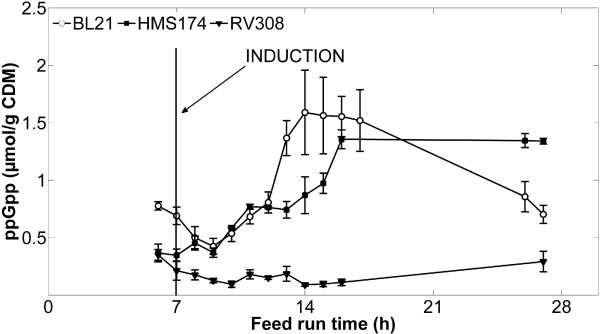
**Analysis of the nucleotide ppGpp.** Levels of ppGpp were analyzed by HPLC during fed-batch cultivations of *E. coli* HMS174, BL21, and RV308 [25]. Data shown are means ± standard error of the mean. Recombinant protein synthesis was induced with IPTG at feed-hour seven.

During the cultivations, we measured the nucleotide ppGpp, an effector molecule that is correlated to the host cell metabolic load imposed by recombinant protein production, and that reflects the metabolic stress level of the cells [24-26]. BL21 and HMS174 exhibited rapid rises in ppGpp concentration shortly after induction (Figure 5). In contrast, RV308 showed no post-induction change in ppGpp level. The ppGpp level of BL21 decreased again at the end of the cultivations.

## Discussion

### Cellular growth

The course of CDM and the final process outcome of the three strains were significantly influenced by the induction strategy and the individual responses of the strains to high-level recombinant protein synthesis. Before induction, all three strains exhibited exponential growth behavior according to the feed media addition (glucose) and the individual yield coefficients. After induction, cell growth was instantly inhibited during cultivations of BL21 and HMS174. In contrast, cell growth of RV308 was inhibited four hours after induction. These results indicate a higher metabolic load of BL21 and HMS174, which was supported by the increasing ppGpp levels in these two strains compared to the constant level observed in RV308. The ppGpp synthesis pathway of RV308 is considered functional, since this strain produced higher levels of ppGpp in another experiment under different conditions.

In the later stage of the process, RV308 and BL21 resumed growth, while the growth rate of HMS174 dropped to zero and did not recommence. Plasmid instability and consequent formation of a non-producing cell population was identified as a reason for the recovery of growth in RV308 and BL21. Koch-plating results showed decreased plasmid-carrying cells at the process end, with only 20% of plasmid-bearing cells remaining in RV308 and none in BL21. However, the analytical method used tends to underestimate the fraction of plasmid-carrying cells because producing cells possess reduced ability to divide or form colonies on agar plates as they enter a “viable but non-culturable” state [27]. Nevertheless, high plasmid loss clearly occurred in BL21 and RV308, while HMS174 demonstrated very high system stability. These results were further confirmed by low plasmid copy numbers of RV308 and BL21, as this value represents the average number for the whole cell population. The changes in cell morphology between non-growing plasmid-harboring and growing plasmid-free cells were not sufficiently distinct to enable differentiation via FACS measurement. It is possible that the formation of plasmid-free non-producing cells was due to plasmid multimer formation, resulting in hampered distribution of plasmids to daughter cells. Multimer formation is a *recA*-dependent phenomenon [28], and genotyping revealed that BL21 and RV308 are both *recA-*positive *(recA*^+^), in contrast to HMS174.

Carbon balancing yielded unexpected results; in the worst-case (phase III of HMS174 cultivations), almost 54% of carbon was not detectable by the applied analytical methods. Protein precipitation experiments showed a significant portion of soluble protein in the supernatant, and the SDS-PAGE pattern of the supernatant revealed high similarity to cell lysate patterns (Additional file 1). Although no quantitative data is available, it is likely that the missing carbon in the calculated C-balances was incorporated in cellular protein DNA and RNA released to the supernatant by cell lysis. The FACS results did not show significant accumulation of dead cells over the entire process time (Figure 4A), even in HMS174; however, this finding does not contradict the assumption of cell lysis, as the FACS method is based on particle counting and thus cannot detect a completely lysed cell. Our results showed that carbon balancing and quantification of missing C may be a method for indirect quantification of cell lysis. Cell lysis could be further quantified by measuring protein or DNA in the supernatant respective of total organic carbon and nitrogen.

### Recombinant protein formation and solubility

The specific amounts of total recombinant protein (including both soluble and insoluble SOD) were comparable in BL21 and HMS174, while RV308 yielded lower amounts (Figure 2A). This difference can be attributed to the significantly reduced levels of SOD accumulated in inclusion bodies in RV308, as the soluble SOD fractions were similar in all three hosts. A maximum of approximately 80 mg/g soluble SOD was obtained in all three strains, indicating a limited cellular capacity for producing soluble SOD under the given cultivation/induction conditions without any influence of the different genetic backgrounds of *E. coli* K–12 or B strains applied (Figure 2). This finding demonstrated that frequently described advantages of *E. coli* BL21 are limited to batch cultivations and that the range of host strains which can be employed in large scale production is significantly broadened by commonly applied carbon limited fed-batch cultivation conditions. At the beginning of the production phase, exclusively soluble SOD was produced; however, over the course of the experiments, more and more protein was accumulated in the insoluble form as IB. In all tested strains, correct SOD folding occurred with a rate of approximately zero, indicating that either the cellular folding machinery became overstrained or essential compounds of the folding infrastructure were no longer available in sufficient amounts. Irrespective of the intrinsic sequence features of SOD that can impact folding behavior, it can be assumed that protease activity partially contributed to the above-described phenomenon, as soluble protein is more exposed to proteolysis. At cultivation end (feed-hour 28) a reduction of the total soluble protein fraction for BL21 and HMS174 in comparison to feed-hour 16 was identified. This can possibly be connected to a potential proteolytic activity. Therefore, the optimal process end-point to achieve maximum yields of the recombinant product was reached at about one generation (seven to eight hours) past induction. No such degradation was identified for RV308 and the amount of total soluble protein even increased by about 10%. This proposes a lower proteolytic activity as the already produced soluble protein stayed approximately constant until the end of the cultivation.

## Conclusions

The present comprehensive comparison of three industrially relevant host strains under production conditions enabled identification of individual benefits and drawbacks of each host cell line, as well as weaknesses of the utilized process conditions. All three hosts were unable to maintain their growth rate after induction because the recombinant gene expression rate exceeded the physiologically tolerable limits. *E. coli* BL21 and RV308 later recovered their cell growth, likely due to plasmid loss. In contrast, *E. coli* HMS174 did not resume cell growth due to its apparent higher system stability. In terms of productivity, BL21 and HMS174 produced larger specific amounts of SOD than RV308; however, all three strains produced similar amounts of soluble SOD. Overall, RV308 showed no degradation of already produced soluble protein, but HMS174 would be the candidate of choice concerning plasmid stability and system integrity. The carbon balancing results revealed significant amounts of non-traceable carbon in all strains, showing that cell lysis is much more pronounced than expected and possibly explaining the phenomena of passive diffusion as lysis [29].

In general, apart from plasmid stability, it was surprising that the differences between the three hosts, especially between *E. coli* B and K–12, were not more pronounced. In particular, RV308 showed high resemblance to the behavior of the B strain. This is possibly connected with the controlled conditions in a bioreactor with pH-regulation and oxygen supply in contrast to standard shake flasks, where the different acetate metabolism of the B strain might be beneficial. The difference in the stability of the plasmid is most likely connected to the distinct *recA* genotypes and proposes a possible knock-out strategy of this gene to enhance plasmid stability.

Based on the presented results, it can be concluded that cultivation temperature and induction level exert a high impact on cellular folding capacity and protein aggregation. The applied conditions, which were close to standard industrial strategies, clearly triggered extremely high metabolic load levels and are therefore suboptimal for achieving the highest possible protein yields. General optimization strategies, such as reduction of cultivation temperature and induction strength, would be applicable for enhancing the soluble recombinant protein fraction in all three hosts. For HMS174, the transcription tuning concept [30] could also be used, while the reduced system stability of BL21 and RV308 would interfere with this optimization approach. The present data should be taken into consideration in future development of new recombinant production processes with any of the three strains (or related hosts). This information will enable improved process predictability, and thereby also substantially shorten process development.

## Methods

All chemicals were purchased from VWR unless otherwise stated. Fed-batch cultivations with a growth rate of 0.1 h^-1^ were performed to evaluate differences in the responses of three *E. coli* hosts to recombinant protein expression. Upon depletion of the glucose from the batch process, an exponential substrate feed was started to maintain a constant growth rate of 0.1 h^-1^ during four doubling times. Recombinant gene expression was induced by a single pulse of IPTG (20 μmol/g CDM after one doubling past the feed start).

### Bacterial strains and plasmid

Experiments were performed with *E. coli* HMS174(DE3)(pET30aSOD), *E. coli* RV308(DE3)(pET30aSOD), and *E. coli* BL21(DE3)(pET30aSOD). RV308 was purchased from the American Type Culture Collection (ATCC # 31608); BL21(DE3) and HMS174(DE3) from Novagen. The DE3-derivative of *E. coli* RV308 was prepared using the λDE3-lysogenization kit (Novagen, Germany) according to the manufacturer’s protocol. The three strains were transformed with the pET30a vector (Novagen, Germany; pET System manual, 11th edition) carrying the model protein human Cu/Zn superoxide dismutase (SOD) (EC 1.15.1.1). This (pET30aSOD) was derived by subcloning of the *sod* gene from (pET11aSOD), described elsewhere [20]. SOD is a highly soluble 32-kDa protein comprising two homologous subunits (a 153-amino acid monomer), which is expressed in the cytoplasm and is nontoxic to the host cell. The T7 expression system is based on expression of the RNA polymerase of bacteriophage T7 under control of the *lac*UV5 in *E. coli *[8,9].

### Cell cultivation

The minimal medium used for cultivations contained 3 g KH_2_PO_4_ and 6 g K_2_HPO_4_∙3H_2_O per liter; these concentrations provided the required buffer capacity and served as sources of P and K. The other components were added in relation to the theoretical grams of CDM to be produced (calculated for 22.5 g in batch-phase and 337.5 g CDM in feed-phase, based on the constant glucose yield coefficient Y_X/S_ of 0.3 g/g): 0.25 g sodium citrate (trisodium salt∙2H_2_O; ACROS organics), 0.10 g MgSO_4_∙7H_2_O, 0.02 g CaCl_2_∙2H_2_O, 50 μL trace element solution, and 3 g glucose∙H_2_O. The trace element solution was prepared in 5 N HCl and included 40 g/L FeSO_4_∙7H_2_O, 10 g/L MnSO_4_∙H_2_O, 10 g/L AlCl_3_∙6H_2_O, 4 g/L CoCl_2_ (Fluka), 2 g/L ZnSO_4_∙7H_2_O, 2 g/L Na_2_MoO_2_∙2H_2_O, 1 g/L CuCl_2_∙2H_2_O, and 0.5 g/L H_3_BO_3_. We also added 4 mg CuCl_2_∙2H_2_O and 3.2 mg ZnSO_4_∙7H_2_O per g CDM. To accelerate initial growth of the population, the complex component yeast extract (0.15 g per g theoretical CDM) was added to the minimal medium to obtain the batch medium. Eight liters of minimal media were prepared for the feeding phase, according to the amount of biological dry matter to be produced (337.5 g); phosphate salts were again added per liter. Nitrogen level was maintained by adding 25% ammonium hydroxide solution (w/w) (MERCK) for pH control.

The cells were grown in a 20-L (14-L working volume, 4-L batch volume) computer-controlled bioreactor (MBR; Wetzikon, CH) equipped with standard control units (Siemens PS7, Intellution iFIX). The pH was maintained at 7.0 ± 0.05 by addition of 25% ammonium hydroxide solution (w/w) (MERCK), the temperature was set to 37°C ± 0.5°C. To avoid oxygen limitation, the dissolved oxygen level was stabilized at above 30% saturation using stirrer speed and aeration rate control. Foaming was suppressed by addition of 0.5 mL antifoam (PPG 2000, Sigma Aldrich) per liter media. To ensure reproducible processes and data, three replicate cultivations were conducted for each strain. Mean values, including standard error of the mean, were used for replicate characterization.

For inoculation, a deep-frozen (-80°C) working cell bank vial was thawed, and 1 mL (optical density OD_600_ = 1) was aseptically transferred to the bioreactor with 30 mL physiologic salt solution. Feeding was started when the culture entered stationary phase. A fed-batch regime with an exponential substrate feed was used to provide a constant growth rate of 0.1 h^-1^ over four doubling times. The substrate feed was controlled by increasing pump speed according to the exponential growth algorithm, X = X_0_ ∙ e^μt^, with superimposed feedback control of weight loss in the substrate tank. Expression system induction was performed by adding isopropyl-β-D-thiogalactoside (IPTG) (GERBU Biotechnik, Germany) to the reactor at 20 μmol IPTG per g calculated CDM (360 g CDM).

### Sample preparation

Optical density at 600 nm was measured with a spectrophotometer (Amersham Biosciences Ultrospec 500 pro). CDM was determined by centrifugation of 10 mL of cell suspension. The supernatant was transferred to an Eppendorf vial, frozen at -20°C, and analyzed by high-performance liquid chromatography (1100 HPLC, Agilent Technologies) using an Aminex HPX-87H ion exclusion column (Biorad), 0.01 N H_2_SO_4_ (20°C and 0.45 mL/minute) as mobile phase and an UV/visible-light (Knauer) and refractive index detectors (Beckmann).

The cells were washed with 7 mL distilled water, resuspended, and transferred to a pre-dried and -weighed beaker, which was then dried at 105°C for 24 h and re-weighed. For the determination of the nucleotide ppGpp, samples were prepared as previously described [25], and quantified by ion-pair reversed-phase HPLC.

### Determination of plasmid-bearing cells by Koch-plating

Plasmid-containing cells were determined by cultivation of bacterial cells using the plasmid-borne antibiotic resistance for selection. Colony forming units (cfu) were counted after 24 h of cultivation on nutrient broth agar plates containing 50 mg/L kanamycin to select for cells containing the vector. To confirm that plasmid-carrying cells over-produced recombinant protein, we also determined the cfu after recombinant gene induction by IPTG (200 mg/L) on selective plates.

### Flow cytometry

Total cell number (TCN) and number of dead cells (DC) were determined by spiking an aliquot of the OD_600_ sample with a known quantity of fluorescent latex counting beads (Becton Dickinson), and staining with fluorescent dyes. The total cell number was recalculated based on the number of hits and the ratio between gated cells and gated counting beads.

Flow cytometry was performed with a FACS Calibur (Becton Dickinson) four-color flow cytometer equipped with a 488-nm laser and the standard filter setup. The CellQuestTM Pro (version 4.1) software package was used for data acquisition and analysis. For preparation of 10 mL staining solution, we mixed 9985 μL sterile filtered 1× PBS buffer (DULBECCO, Biochrom AG), 5 μL Tween20 (ACROS Chemicals), and 50 μL propidium iodide stock solution (1 mg/mL in distilled water; Becton Dickinson). This solution was aliquoted into FACS-tubes, 500 μL each. For determination of TCN via flow cytometry, 25 μL of the OD_600_ sample (1:101 or 1:201 dilution) was added to the staining solution in the FACS-tube. These dilution steps resulted in a counting rate of between 3000 and 6000 events per second. TCN was determined via ratiometric counting, taking into account the number of added counting beads, the ratio of sample per counting bead, and the sample volume and dilution factors according to the following equation:

$$\begin{array}{ll} \mathrm{TCN} & =\frac{\text{Cell}-\text{quantity}\left(\text{region}\right)}{\text{Bead}-\text{quantity}\left(\text{region}\right)}\\ & \;*\frac{\text{bead}-\text{concentration}\left(\mathrm{\mu l}\right)}{\text{sample}-\text{volume}\left(\text{μl}\right)}*\text{dilution}-\text{factor} \end{array}$$

Cell viability was determined based on the dye-discharging capacity of living cells, using a staining protocol with a simple live/dead discrimination via PI [31]. Propidium iodide binds to double-strand DNA, but cannot penetrate the intact cell membrane of *E. coli*. Bacterial cells showing PI fluorescence (Ex = 535 nm, Em = 617 nm) can be assumed to be dead, but morphologically intact. The number of dead cells was calculated by the ratio of total cell number and PI-positive cells. Viable cell number (VCN) was calculated as $\mathrm{VCN}=\mathrm{TCN}-\frac{\mathrm{TCN}*\mathrm{DC}}{100}$

### Product analysis

The recombinant protein was quantified via SOD-ELISA [32], and the ratio between soluble protein and inclusion bodies was determined by SDS-PAGE as previously described [33].

### On-line measurements

To determine base consumption, ammonia consumption was measured by recording the changes in weight. The O_2_ and CO_2_ contents in the outlet air were determined using a Hartmann and Braun Advanced Optima gas analyzer [34,35].

## Competing interests

The authors declare that they have no competing interests.

## Authors’ contributions

KM and ML planned and performed the cultivations. KM analyzed the data, and wrote the manuscript. MC-P supervised and directed the off-line analytics (ELISA, SDS-PAGE, and HPLC). KM, GS, and KB participated in research design and discussion. GS drafted the manuscript and directed the research. All authors read and approved the final manuscript.

## Supplementary Material

Additional file 1 Time-course SDS-PAGE analysis of the soluble and insoluble protein fractions of all fed-batch cultivations analyzed in this study. Click here for file
