# Supplementary material for: Evaluation of three industrial Escherichia coli strains in fed-batch cultivations during high-level SOD protein production
Source: Microb Cell Fact. 2013 Jun 11;12:58. doi: 10.1186/1475-2859-12-58 (PMC3698069; doi:10.1186/1475-2859-12-58)
Supplement: Additional file 1 — Time-course SDS-PAGE analysis of the soluble and insoluble protein fractions of all fed-batch cultivations analyzed in this study. [file 1475-2859-12-58-S1.pdf]

fermentation	host	tab-file	samples	feed-hour
SOD8	BL21(DE3)(pET30a_hSOD)	BL21_SOD8a	9-13	8-12
		BL21_SOD8b	14-18	13-17
SOD9	BL21(DE3)(pET30a_hSOD)	BL21_SOD9a	9-13	8-12
		BL21_SOD9b	14-18	13-17
SOD10	BL21(DE3)(pET30a_hSOD)	BL21_SOD10a	9-13	8-12
		BL21_SOD10b	14-18	13-17

Ü	soluble fraction
IB	insoluble fraction

fermentation	host	tab-file	samples	feed-hour
SOD12	HMS174(DE3)(pET30a_hSOD)	HMS174_SOD12a	9-13	8-12
	HMS174(DE3)(pET30a_hSOD)	HMS174_SOD12b	14-18	13-17
SOD13	HMS174(DE3)(pET30a_hSOD)	HMS174_SOD13a	9-13	8-12
	HMS174(DE3)(pET30a_hSOD)	HMS174_SOD13b	14-17	13-16
SOD14	HMS174(DE3)(pET30a_hSOD)	HMS174_SOD14a	9-13	8-12
	HMS174(DE3)(pET30a_hSOD)	HMS174_SOD14b	14-17	13-16
SOD12,13,14	HMS174(DE3)(pET30a_hSOD)	HMS174-SOD12-14WH	repeats of different samples	

Ü	soluble fraction
IB	insoluble fraction

fermentation	host	tab-file	samples	feed-hour
SOD22	RV308(DE3)(pET30a_hSOD)	RV308_SOD22a	9-12,14	8-11,13
	RV308(DE3)(pET30a_hSOD)	RV308_SOD22b	16-17	15-16
SOD24	RV308(DE3)(pET30a_hSOD)	RV308_SOD24a	9-13	8-12
	RV308(DE3)(pET30a_hSOD)	RV308_SOD24b	14-17	13-16
SOD25	RV308(DE3)(pET30a_hSOD)	RV308_SOD25a	9-13	8-12
	RV308(DE3)(pET30a_hSOD)	RV308_SOD25b	14-17	13-16

Ü	soluble fraction
IB	insoluble fraction

NuPAGE Elektrophorese

Datum: 24.07.2008

Versuch SOD 8 - Verteilung IBs / lösl. Protein

Operator Conni

Gel Nu PAGE 4-12% Bis Tris Gel
MES running buffer

Marker Mark 12™

Settings 200 V, max. 400 mA, 30´-50´

Scan-Datei 24072008_SOD8a

Bemerkungen

well	Probe	red	Vol [µl]	comments
1	MW Standard Mark 12		7	
2	SOD Standard (0.196 mg/ml)	+	15	
3	SOD 8 / 9 Ü	+	15	
4	SOD 8 / 9 IB	+	15	
5	SOD 8 / 10 Ü	+	15	
6	SOD 8 / 10 IB	+	15	
7	SOD 8 / 11 Ü	+	15	
8	SOD 8 / 11 IB	+	15	
9	SOD 8 / 12 Ü	+	15	
10	SOD 8 / 12 IB	+	15	
11	SOD 8 / 13 Ü	+	15	
12	SOD 8 / 13 IB	+	15	

Quantifizierung

well	Probe	Vol [µl]	raw vol.	ratio [%]	comments
1	MW Standard Mark 12	7			
2	SOD Standard (0.196 mg/ml)	15	54536582.7		
3	SOD 8 / 9 Ü	15	103073586	94.2	
4	SOD 8 / 9 IB	15	6334798.5	5.8	
5	SOD 8 / 10 Ü	15	108017233	93.8	
6	SOD 8 / 10 IB	15	7152027.31	6.2	
7	SOD 8 / 11 Ü	15	145300957		
8	SOD 8 / 11 IB	15			
9	SOD 8 / 12 Ü	15	137468378	56.2	
10	SOD 8 / 12 IB	15	107303540	43.8	
11	SOD 8 / 13 Ü	15	117656303	45.6	
12	SOD 8 / 13 IB	15	140534767	54.4	

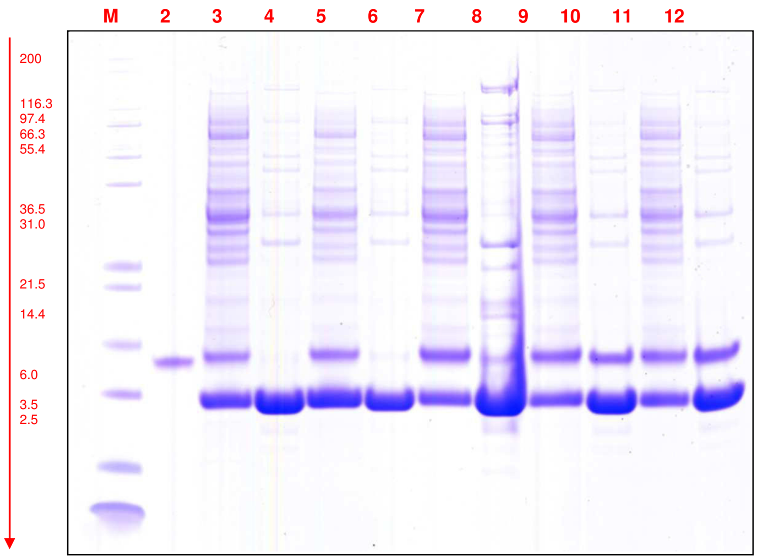

NuPAGE Elektrophorese

Datum: 24.07.2008

Versuch SOD 8 - Verteilung IBs / lösl. Protein
Operator Conni
Gel Nu PAGE 4-12% Bis Tris Gel
MES running buffer
Marker Mark 12™
Settings 200 V, max. 400 mA, 30´-50´
Scan-Datei 24072008_SOD8b

Bemerkungen

well	Probe	red	Vol [µl]	comments
1	MW Standard Mark 12		7	
2	SOD Standard (0.196 mg/ml)	+	15	
3	SOD 8 / 14 Ü	+	15	
4	SOD 8 / 14 IB	+	15	
5	SOD 8 / 15 Ü	+	15	
6	SOD 8 / 15 IB	+	15	
7	SOD 8 / 16 Ü	+	15	
8	SOD 8 / 16 IB	+	15	
9	SOD 8 / 17 Ü	+	15	
10	SOD 8 / 17 IB	+	15	
11	SOD 8 / 18 Ü	+	15	
12	SOD 8 / 18 IB	+	15	

Quantifizierung

well	Probe	Vol [µl]	raw vol.	ratio [%]	comments
1	MW Standard Mark 12	7			
2	SOD Standard (0.196 mg/ml)	15	39603237		
3	SOD 8 / 14 Ü	15	103538181	42.7	
4	SOD 8 / 14 IB	15	138675114	57.3	
5	SOD 8 / 15 Ü	15	102832098	41.5	
6	SOD 8 / 15 IB	15	145213550	58.5	
7	SOD 8 / 16 Ü	15	106742342	40.4	
8	SOD 8 / 16 IB	15	157497757	59.6	
9	SOD 8 / 17 Ü	15	109745483	40.8	
10	SOD 8 / 17 IB	15	159545860	59.2	
11	SOD 8 / 18 Ü	15	118700619	58.0	
12	SOD 8 / 18 IB	15	86065309.8	42.0	

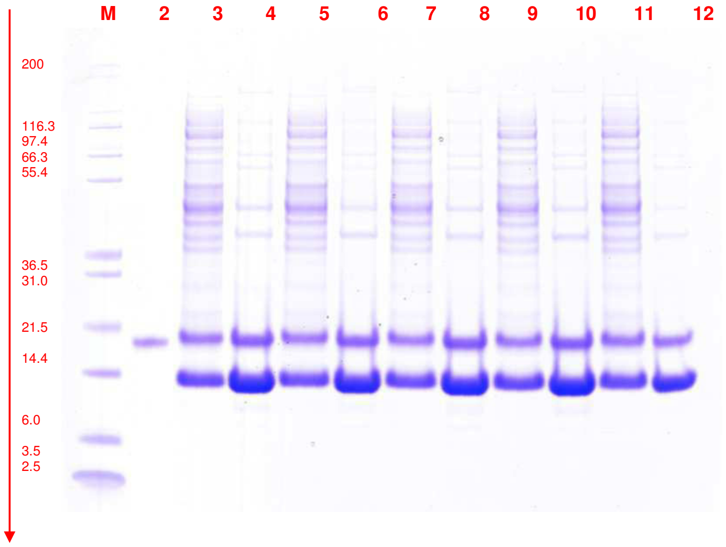

	1.14E+08
	1.24E+08
106742342	1.07E+08
157497757	1.39E+08
109745483	1.1E+08
159545860	1.48E+08

NuPAGE Elektrophorese

Datum: 23.07.2008

Versuch SOD 9 - Verteilung IBs / lösl. Protein

Operator Conni + Moni

Gel Nu PAGE 4-12% Bis Tris Gel
MES running buffer

Marker Mark 12™

Settings 200 V, max. 400 mA, 30´-50´

Scan-Datei 23072008a

Bemerkungen

Quantifizierung

well	Probe	red	Vol [µl]	comments
1	MW Standard Mark 12		7	
2	SOD Standard (0.196 mg/ml)	+	15	
3	SOD 9 / 9 Ü	+	15	
4	SOD 9 / 9 IB	+	15	
5	SOD 9 / 10 Ü	+	15	
6	SOD 9 / 10 IB	+	15	
7	SOD 9 / 11 Ü	+	15	
8	SOD 9 / 11 IB	+	15	
9	SOD 9 / 12 Ü	+	15	
10	SOD 9 / 12 IB	+	15	
11	SOD 9 / 13 Ü	+	15	
12	SOD 9 / 13 IB	+	15	

well	Probe	Vol [µl]	raw vol.	ratio [%]	comments
1	MW Standard Mark 12	7			
2	SOD Standard (0.196 mg/ml)	15	177125942		
3	SOD 9 / 9 Ü	15	141124354	91.7	
4	SOD 9 / 9 IB	15	12831239	8.3	
5	SOD 9 / 10 Ü	15	239007687	86.0	
6	SOD 9 / 10 IB	15	38802117	14.0	
7	SOD 9 / 11 Ü	15	299144402	64.1	
8	SOD 9 / 11 IB	15	167896815	35.9	
9	SOD 9 / 12 Ü	15	277814430	52.2	
10	SOD 9 / 12 IB	15	254657959	47.8	
11	SOD 9 / 13 Ü	15	277838331	47.1	
12	SOD 9 / 13 IB	15	312102085	52.9	

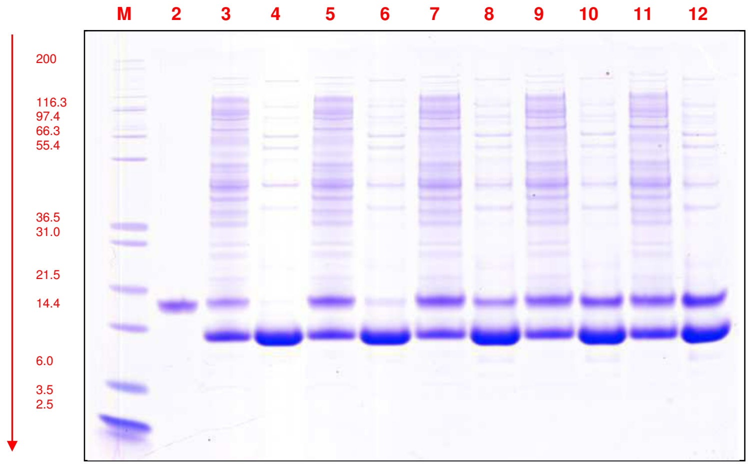

NuPAGE Elektrophorese

Datum: 23.07.2008

Versuch SOD 9 - Verteilung IBs / lösl. Protein

Operator Conni + Moni

Gel Nu PAGE 4-12% Bis Tris Gel
MES running buffer

Marker Mark 12™

Settings 200 V, max. 400 mA, 30´-50´

Scan-Datei 23072008

Bemerkungen

well	Probe	red	Vol [µl]	comments
1	MW Standard Mark 12		7	
2	SOD Standard (0.196 mg/ml)	+	15	
3	SOD 9 / 14 Ü	+	15	
4	SOD 9 / 14 IB	+	15	
5	SOD 9 / 15 Ü	+	15	
6	SOD 9 / 15 IB	+	15	
7	SOD 9 / 16 Ü	+	15	
8	SOD 9 / 16 IB	+	15	
9	SOD 9 / 17 Ü	+	15	
10	SOD 9 / 17 IB	+	15	
11	SOD 9 / 18 Ü	+	15	
12	SOD 9 / 18 IB	+	15	

Quantifizierung

well	Probe	Vol [µl]	raw vol.	ratio [%]	comments
1	MW Standard Mark 12	7			
2	SOD Standard (0.196 mg/ml)	15	24325653.1		
3	SOD 9 / 14 Ü	15	137237549	40.9	
4	SOD 9 / 14 IB	15	197957654	59.1	
5	SOD 9 / 15 Ü	15	137383698	41.1	
6	SOD 9 / 15 IB	15	197090324	58.9	
7	SOD 9 / 16 Ü	15	137642851	39.9	
8	SOD 9 / 16 IB	15	207165746	60.1	
9	SOD 9 / 17 Ü	15	101910707	33.8	
10	SOD 9 / 17 IB	15	199310353	66.2	
11	SOD 9 / 18 Ü	15	132908960	41.3	
12	SOD 9 / 18 IB	15	188874257	58.7	

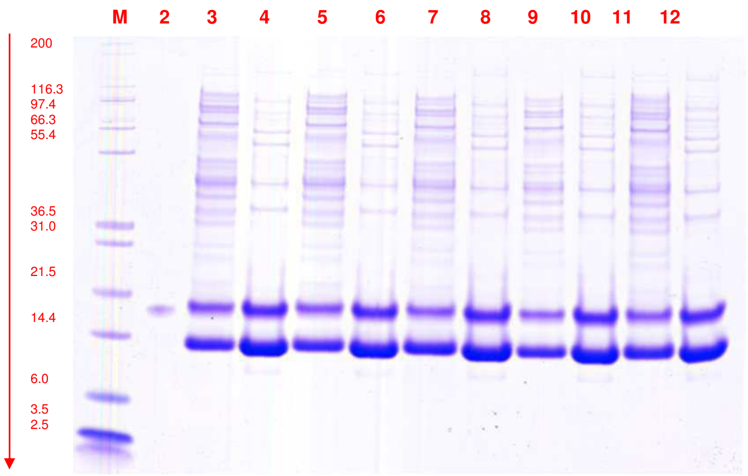

NuPAGE Elektrophorese

Datum: 28.07.2008

Versuch SOD 10 - Verteilung IBs / lösl. Protein

Operator Conni

Gel Nu PAGE 4-12% Bis Tris Gel
MES running buffer

Marker Mark 12™

Settings 200 V, max. 400 mA, 30´-50´

Scan-Datei 28072008_SOD10a

Bemerkungen

Quantifizierung

well	Probe	red	Vol [µl]	comments
1	MW Standard Mark 12		7	
2	SOD Standard (0.196 mg/ml)	+	15	
3	SOD 10 / 9 Ü	+	15	
4	SOD 10 / 9 IB	+	15	
5	SOD 10 / 10 Ü	+	15	
6	SOD 10 / 10 IB	+	15	
7	SOD 10 / 11 Ü	+	15	
8	SOD 10 / 11 IB	+	15	
9	SOD 10 / 12 Ü	+	15	
10	SOD 10 / 12 IB	+	15	
11	SOD 10 / 13 Ü	+	15	
12	SOD 10 / 13 IB	+	15	

well	Probe	Vol [µl]	raw vol.	ratio [%]	comments
1	MW Standard Mark 12	7			
2	SOD Standard (0.196 mg/ml)	15	254619		
3	SOD 10 / 9 Ü	15	318052	93.1	
4	SOD 10 / 9 IB	15	23471	6.9	
5	SOD 10 / 10 Ü	15	380994	85.9	
6	SOD 10 / 10 IB	15	62578	14.1	
7	SOD 10 / 11 Ü	15	456192	72.2	
8	SOD 10 / 11 IB	15	175401	27.8	
9	SOD 10 / 12 Ü	15	409941	51.2	
10	SOD 10 / 12 IB	15	391494	48.8	
11	SOD 10 / 13 Ü	15	354933	47.5	
12	SOD 10 / 13 IB	15	392134	52.5	

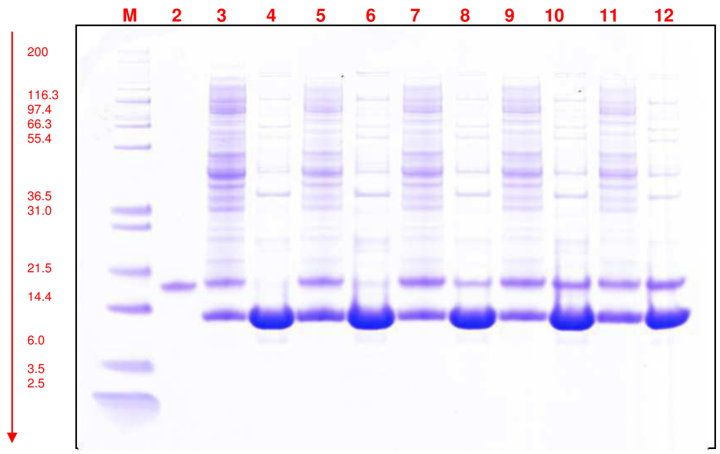

NuPAGE Elektrophorese

Datum: 28.07.2008

Versuch SOD 10 - Verteilung IBs / lösl. Protein

Operator Conni

Gel Nu PAGE 4-12% Bis Tris Gel
MES running buffer

Marker Mark 12™

Settings 200 V, max. 400 mA, 30´-50´

Scan-Datei 28072008_SOD10b

Bemerkungen

well	Probe	red	Vol [µl]	comments
1	MW Standard Mark 12		7	
2	SOD Standard (0.196 mg/ml)	+	15	
3	SOD 10 / 14 Ü	+	15	
4	SOD 10 / 14 IB	+	15	
5	SOD 10 / 15 Ü	+	15	
6	SOD 10 / 15 IB	+	15	
7	SOD 10 / 16 Ü	+	15	
8	SOD 10 / 16 IB	+	15	
9	SOD 10 / 17 Ü	+	15	
10	SOD 10 / 17 IB	+	15	
11	SOD 10 / 18 Ü	+	15	
12	SOD 10 / 18 IB	+	15	

Quantifizierung

well	Probe	Vol [µl]	raw vol.	ratio [%]	comments
1	MW Standard Mark 12	7			
2	SOD Standard (0.196 mg/ml)	15	188008.16		
3	SOD 10 / 14 Ü	15	302576.52	42.3	
4	SOD 10 / 14 IB	15	413331.84	57.7	
5	SOD 10 / 15 Ü	15	272707.82	35.3	
6	SOD 10 / 15 IB	15	499474.01	64.7	
7	SOD 10 / 16 Ü	15	282609.94	37.1	
8	SOD 10 / 16 IB	15	478792.25	62.9	
9	SOD 10 / 17 Ü	15	251752.8		
10	SOD 10 / 17 IB	15			
11	SOD 10 / 18 Ü	15			
12	SOD 10 / 18 IB	15			

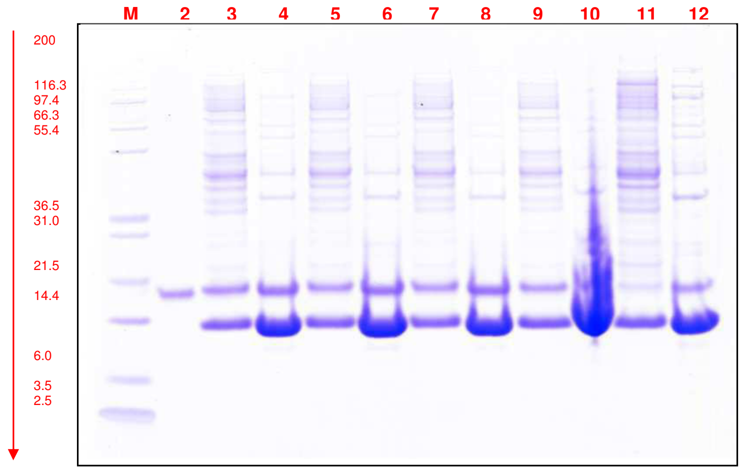

NuPAGE Elektrophorese

Datum: 23.09.2008

Versuch SOD 12 - Verteilung IBs / lösl. Protein - versch. Aufschlüsse

Operator Conni

Gel Nu PAGE 4-12% Bis Tris Gel
MES running buffer

Marker Mark 12™

Settings 200 V, max. 400 mA, 30´-50´

Scan-Datei 23092008b

Bemerkungen

Quantifizierung

well	Probe	red	Vol [µl]	comments
1	MW Standard Mark 12		7	
2	SOD 12/8 Ü	+	15	
3	SOD 12/8 IB	+	15	
4	SOD 12/9 Ü	+	15	
5	SOD 12/9 IB	+	15	
6	SOD 12/10 Ü	+	15	
7	SOD 12/10 IB	+	15	
8	SOD 12/11 Ü	+	15	
9	SOD 12/11 IB	+	15	
10	SOD 12/12 Ü	+	15	
11	SOD 12/12 IB	+	15	
12	SOD Standard (0.196 mg/ml)	+	15	

well	Probe	Vol [µl]	raw vol.	ratio [%]	comments
1	MW Standard Mark 12	7			
2	SOD 12/8 Ü	15	0	0	
3	SOD 12/8 IB	15	0	0	
4	SOD 12/9 Ü	15	32042	100	
5	SOD 12/9 IB	15	0	0	
6	SOD 12/10 Ü	15	64075	100	
7	SOD 12/10 IB	15	0	0	
8	SOD 12/11 Ü	15	81461	88	
9	SOD 12/11 IB	15	11478	12	
10	SOD 12/12 Ü	15	65984	59	
11	SOD 12/12 IB	15	46213	41	
12	SOD Standard (0.196 mg/ml)	15			

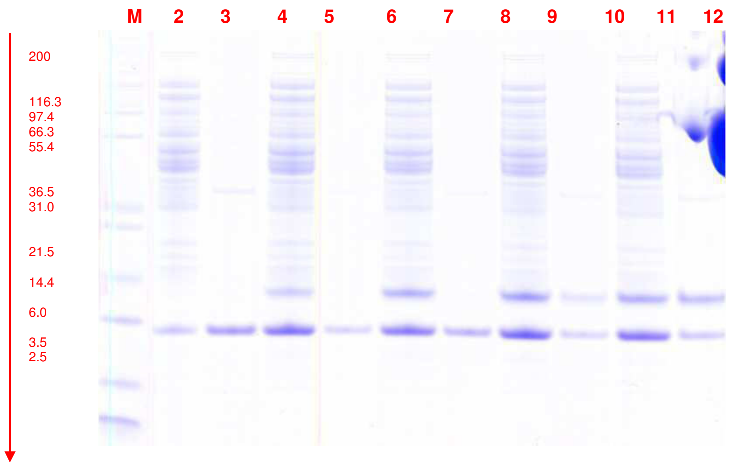

NuPAGE Elektrophorese

Datum: 23.09.2008

Versuch SOD 12 - Verteilung IBs / lösl. Protein - versch. Aufschlüsse

Operator Conni

Gel Nu PAGE 4-12% Bis Tris Gel
MES running buffer

Marker Mark 12™

Settings 200 V, max. 400 mA, 30´-50´

Scan-Datei 23092008a

Bemerkungen

well	Probe	red	Vol [µl]	comments
1	MW Standard Mark 12		7	
2	SOD 12/14 Ü	+	15	
3	SOD 12/14 IB	+	15	
4	SOD 12/13 Ü	+	15	
5	SOD 12/13 IB	+	15	
6	SOD 12/15 Ü	+	15	
7	SOD 12/15 IB	+	15	
8	SOD 12/16 Ü	+	15	
9	SOD 12/16 IB	+	15	
10	SOD 12/17 Ü	+	15	
11	SOD 12/17 IB	+	15	
12	SOD Standard (0.196 mg/ml)	+	15	

Quantifizierung

well	Probe	Vol [µl]	raw vol.	ratio [%]	comments
1	MW Standard Mark 12	7			
2	SOD 12/14 Ü	15	18402	34	
3	SOD 12/14 IB	15	34980	66	
4	SOD 12/13 Ü	15	25942	52	
5	SOD 12/13 IB	15	24259	48	
6	SOD 12/15 Ü	15			
7	SOD 12/15 IB	15	28426		
8	SOD 12/16 Ü	15	9898	24	
9	SOD 12/16 IB	15	31460	76	
10	SOD 12/17 Ü	15	12601	31	
11	SOD 12/17 IB	15	27780	69	
12	SOD Standard (0.196 mg/ml)	15	350932	93	

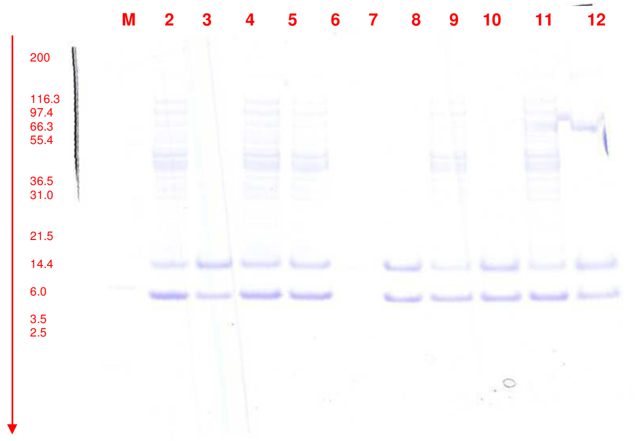

NuPAGE Elektrophorese

Datum: 07.10.2008

Versuch SOD 13 - Verteilung IBs / lösl. Protein

Operator Patricia

Gel Nu PAGE 4-12% Bis Tris Gel
MES running buffer

Marker Mark 12™

Settings 200 V, max. 400 mA, 30´-50´

Scan-Datei 07102008_SOD13a

Bemerkungen

Quantifizierung

well	Probe	red	Vol [µl]	comments
1	MW Standard Mark 12		7	
2	SOD 13 / 9 Ü	+	15	
3	SOD 13 / 9 IB	+	15	
4	SOD 13 / 10 Ü	+	15	
5	SOD 13/ 10 IB	+	15	
6	SOD 13 / 11 Ü	+	15	
7	SOD 13 / 11 IB	+	15	
8	SOD 13 / 12 Ü	+	15	
9	SOD 13 / 12 IB	+	15	
10	SOD 13 / 13 Ü	+	15	
11	SOD 13 / 13 IB	+	15	
12	SOD Standard (0.196 mg/ml)	+	15	

well	Probe	Vol [µl]	position	raw vol.	ratio [%]	comments
1	MW Standard Mark 12	7				
2	SOD 13 / 9 Ü	15	498	10,905,255	100	
3	SOD 13 / 9 IB	15		0	0	
4	SOD 13 / 10 Ü	15	500	18,315,319	100	
5	SOD 13/ 10 IB	15		0	0	
6	SOD 13 / 11 Ü	15	497	20,367,310	86	
7	SOD 13 / 11 IB	15	500	3,299,123	14	
8	SOD 13 / 12 Ü	15	497	19,334,855	62	
9	SOD 13 / 12 IB	15	495	11,652,718	38	
10	SOD 13 / 13 Ü	15	497	15,669,183	44	
11	SOD 13 / 13 IB	15	500	19,729,940	56	
12	SOD Standard (0.196 mg/ml)	15	500	19,456,177		

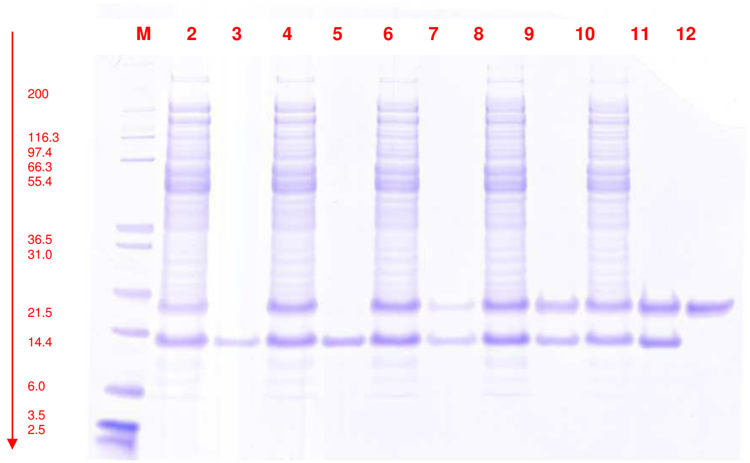

NuPAGE Elektrophorese

Datum: 07.10.2008

Versuch SOD 13 - Verteilung IBs / lösl. Protein, WH SOD 12

Operator Patricia

Gel Nu PAGE 4-12% Bis Tris Gel
MES running buffer

Marker Mark 12™

Settings 200 V, max. 400 mA, 30´-50´

Scan-Datei 07102008-SOD13b

Bemerkungen

Quantifizierung

well	Probe	red	Vol [µl]	comments
1	MW Standard Mark 12		7	
2	SOD 13/14 Ü	+	15	
3	SOD 13/14 IB	+	15	
4	SOD 13/15 Ü	+	15	
5	SOD 13/15 IB	+	15	
6	SOD 13/16 Ü	+	15	
7	SOD 13/16 IB	+	15	
8	SOD 13/17 Ü	+	15	
9	SOD 13/17 IB	+	15	
10	SOD 12/15 IB	+	15	
11	SOD 12/15 Ü	+	15	
12	SOD Standard (0.181 mg/ml)	+	15	

well	Probe	Vol [µl]	position	raw vol.	ratio [%]	comments
1	MW Standard Mark 12	7				
2	SOD 13/14 Ü	15	487	16,062,268	71	
3	SOD 13/14 IB	15	487	6,407,347	29	
4	SOD 13/15 Ü	15	490	15,462,531	36	
5	SOD 13/15 IB	15	489	27,957,832	64	
6	SOD 13/16 Ü	15	484	14,819,829	33	
7	SOD 13/16 IB	15	487	30,471,064	67	
8	SOD 13/17 Ü	15	489	14,547,677	33	
9	SOD 13/17 IB	15	493	29,626,350	67	
10	SOD 12/15 IB	15	490	13,376,199	45	
11	SOD 12/15 Ü	15	490	16,040,081	55	
12	SOD Standard (0.181 mg/ml)	15	489	24,827,720		

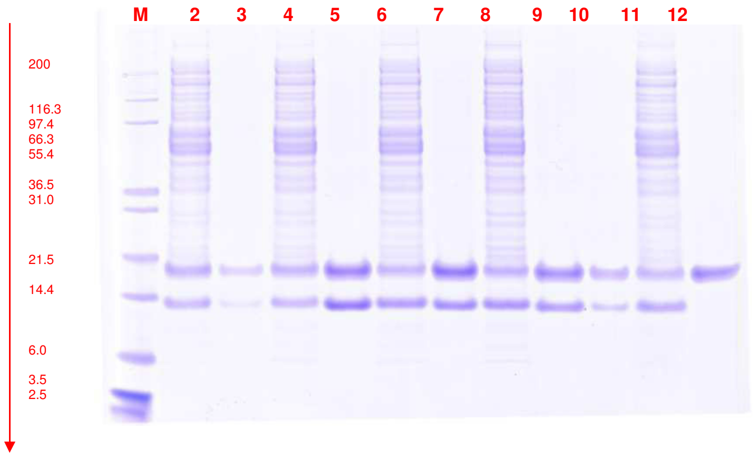

NuPAGE Elektrophorese

Datum: 04.01.1900

Versuch SOD 14- Verteilung IBs / lösl. Protein

Operator Patricia

Gel Nu PAGE 4-12% Bis Tris Gel
MES running buffer

Marker Mark 12™

Settings 200 V, max. 400 mA, 30´-50´

Scan-Datei 04112008_SOD14a

Bemerkungen

Quantifizierung

well	Probe	red	Vol [µl]	comments
1	MW Standard Mark 12		7	
2	SOD 14 / 9 Ü	+	15	
3	SOD 14 / 9 IB	+	15	
4	SOD 14 / 10 Ü	+	15	
5	SOD 14/ 10 IB	+	15	
6	SOD 14 / 11 Ü	+	15	
7	SOD 14 / 11 IB	+	15	
8	SOD 14 / 12 Ü	+	15	
9	SOD 14 / 12 IB	+	15	
10	SOD 14 / 13 Ü	+	15	
11	SOD 14 / 13 IB	+	15	
12	SOD Standard (0.196 mg/ml)	+	15	

well	Probe	Vol [µl]	position	raw vol.	ratio [%]	comments
1	MW Standard Mark 12	7				
2	SOD 14 / 9 Ü	15	421	5,462,167	100	
3	SOD 14 / 9 IB	15		0	0	
4	SOD 14 / 10 Ü	15	419	9,965,516	100	
5	SOD 14/ 10 IB	15		0	0	
6	SOD 14 / 11 Ü	15	425	10,945,944	64	
7	SOD 14 / 11 IB	15	419	6,261,183	36	
8	SOD 14 / 12 Ü	15	423	11,188,707	37	
9	SOD 14 / 12 IB	15	423	18,665,375	63	
10	SOD 14 / 13 Ü	15	425	8,137,881	22	
11	SOD 14 / 13 IB	15	425	29,236,806	78	
12	SOD Standard (0.196 mg/ml)	15			0	

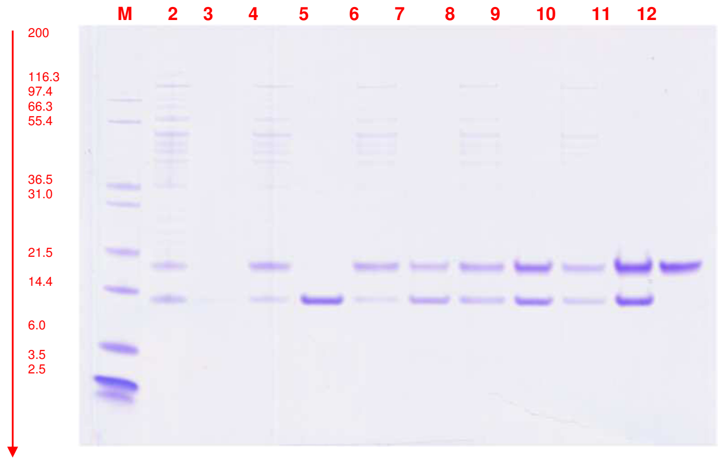

NuPAGE Elektrophorese

Datum: 04.11.2008

Versuch SOD 14 - Verteilung IBs / lösl. Protein, WH SOD 12

Operator Conni

Gel Nu PAGE 4-12% Bis Tris Gel
MES running buffer

Marker Mark 12™

Settings 200 V, max. 400 mA, 30´-50´

Scan-Datei 04112008-SOD14b

Bemerkungen

Quantifizierung

well	Probe	red	Vol [µl]	comments
1	MW Standard Mark 12		7	
2	SOD 14/14 Ü	+	15	
3	SOD 14/14 IB	+	15	
4	SOD 14/15 Ü	+	15	
5	SOD 14/15 IB	+	15	
6	SOD 14/16 Ü	+	15	
7	SOD 14/16 IB	+	15	
8	SOD 14/17 Ü	+	15	
9	SOD 14/17 IB	+	15	
10	SOD 14/14 Ü	+	15	
11	SOD 14/14 IB	+	15	
12	SOD Standard (0.196 mg/ml)	+	15	

well	Probe	Vol [µl]	position	raw vol.	ratio [%]	comments
1	MW Standard Mark 12	7				
2	SOD 14/14 Ü	15	642	114838908	36	
3	SOD 14/14 IB	15	644	201164180	64	
4	SOD 14/15 Ü	15	640	96913415	31	
5	SOD 14/15 IB	15	642	215577254	69	
6	SOD 14/16 Ü	15	646	96499131	30	
7	SOD 14/16 IB	15	644	220976310	70	
8	SOD 14/17 Ü	15	644	92649271	30	
9	SOD 14/17 IB	15	644	215174535	70	
10	SOD 14/14 Ü	15	640	106769622	35	
11	SOD 14/14 IB	15	638	201498280	65	
12	SOD Standard (0.196 mg/ml)	15				

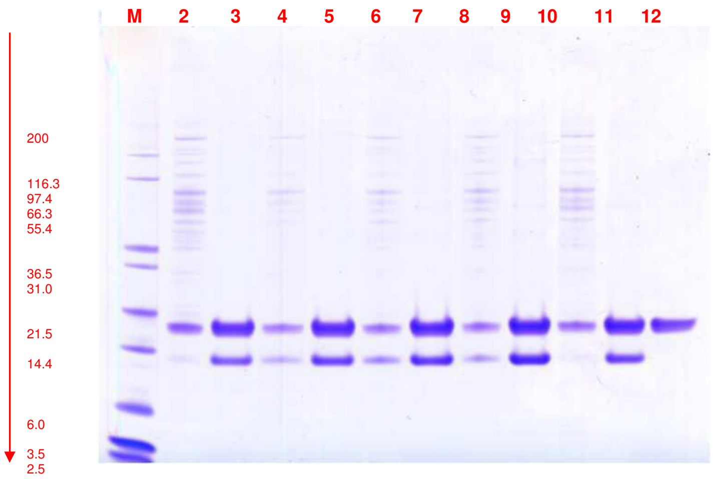

NuPAGE Elektrophorese

Datum: 18.11.2008

Versuch SOD 12, 13, 14 WH - Verteilung IBs / lösl. Protein

Operator Patricia

Gel Nu PAGE 4-12% Bis Tris Gel
MES running buffer

Marker Mark 12™

Settings 200 V, max. 400 mA, 30´-50´

Scan-Datei 18112008b.tif

Bemerkungen

Quantifizierung

well	Probe	red	Vol [µl]	comments
1	MW Standard Mark 12		7	
2	SOD 13/13 Ü	+	15	
3	SOD 13/13 IB	+	15	
4	SOD 13/14 Ü	+	15	
5	SOD 13/14 IB	+	15	
6	SOD 12/15 Ü	+	15	
7	SOD 12/15 IB	+	15	
8	SOD 12/16 Ü	+	15	
9	SOD 12/16 IB	+	15	
10	SOD 14/11 Ü	+	15	
11	SOD 14/11 IB	+	15	
12	SOD Standard (0.181 mg/ml)	+	15	

well	Probe	Vol [µl]	position	raw vol.	ratio [%]	comments
1	MW Standard Mark 12	7				
2	SOD 13/13 Ü	15	336	33,229,964	48	
3	SOD 13/13 IB	15	334	36,623,062	52	
4	SOD 13/14 Ü	15	334	29,355,235	40	
5	SOD 13/14 IB	15	336	43,486,253	60	
6	SOD 12/15 Ü	15	338	27,370,232	36	
7	SOD 12/15 IB	15	340	47,681,893	64	
8	SOD 12/16 Ü	15	336	26,014,549	35	
9	SOD 12/16 IB	15	334	48,516,951	65	
10	SOD 14/11 Ü	15	341	41,213,683	77	
11	SOD 14/11 IB	15	341	12,549,118	23	
12	SOD Standard (0.181 mg/ml)	15	331	32,514,066		

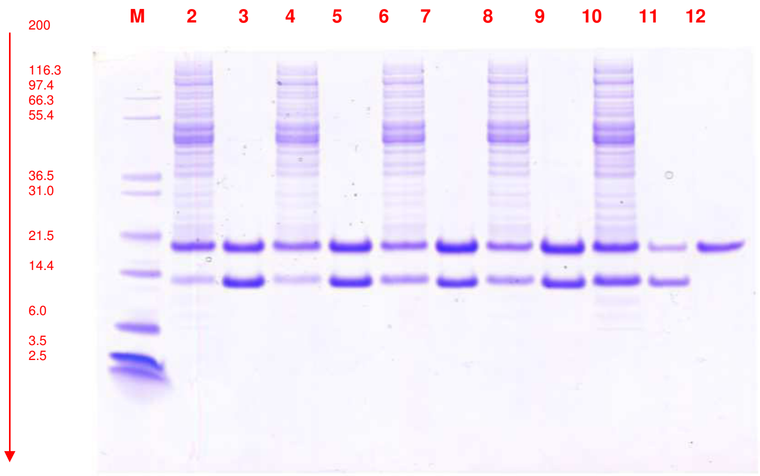

NuPAGE Elektrophorese **Datum:** 23.04.2009 Quantifizierung 23042009_SOD22a

Versuch SOD 22 - Verteilung IBs / lösl. Protein

Operator Patricia

Gel Nu PAGE 4-12% Bis Tris Gel
MES running buffer **Marker** Mark 12™

Settings 200 V, max. 400 mA, 30´-50´ **Scan-Datei** 23042009_SOD22a

Bemerkungen

Quantifizierung

well	Probe	red	Vol [µl]	comments
1	MW Standard Mark 12		7	
2	SOD 22/9 Ü	+	15	
3	SOD 22/9 IB	+	15	
4	SOD 22/10 Ü	+	15	
5	SOD 22/10 IB	+	15	
6	SOD 22/11 Ü	+	15	
7	SOD 22/11 IB	+	15	
8	SOD 22/12 Ü	+	15	
9	SOD 22/12 IB	+	15	
10	SOD 22/14 Ü	+	15	
11	SOD 22/14 IB	+	15	
12	SOD Standard (0.196 mg/ml)	+	15	

well	Probe	Vol [µl]	position	raw vol.	ratio [%]	comments
1	MW Standard Mark 12	7				
2	SOD 22/9 Ü	15	612	13875537	95	
3	SOD 22/9 IB	15	616	773632	5	
4	SOD 22/10 Ü	15	618	16656763	84	
5	SOD 22/10 IB	15	622	3202499	16	
6	SOD 22/11 Ü	15	627	16487258	63	
7	SOD 22/11 IB	15	627	9616875	37	
8	SOD 22/12 Ü	15	627	18972942	54	
9	SOD 22/12 IB	15	622	16234609	46	
10	SOD 22/14 Ü	15	616	16426864	45	
11	SOD 22/14 IB	15	616	19863922	55	
12	SOD Standard (0.196 mg/ml)	15				

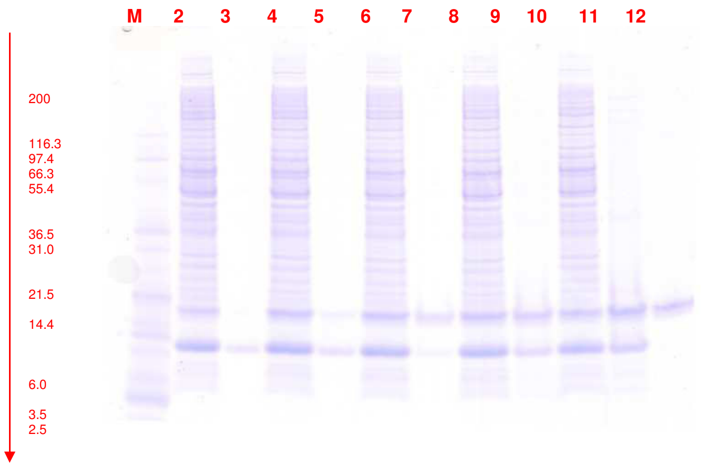

NuPAGE Elektrophorese

Datum: 23.04.2009

Quantifizierung

Versuch SOD 22 + Alex - Verteilung IBs / lösl. Protein

Operator Patricia

Gel Nu PAGE 4-12% Bis Tris Gel
MES running buffer

Marker Mark 12™

Settings 200 V, max. 400 mA, 30´-50´

Scan-Datei 23042009_SOD22b_Alex

Bemerkungen

Quantifizierung

well	Probe	red	Vol [µl]	comments
1	MW Standard Mark 12		7	
2	SOD 22/16 Ü	+	15	
3	SOD 22/16 B	+	15	
4	SOD 22/17 Ü	+	15	
5	SOD 22/17 IB	+	15	
6	divers	+	15	
7	divers	+	15	
8	divers	+	15	
9	divers	+	15	
10	divers	+	15	
11	divers	+	15	
12	SOD Standard (0.196 mg/ml)	+	15	

well	Probe	Vol [µl]	position	raw vol.	ratio [%]	comments
1	MW Standard Mark 12	7				
2	SOD 22/16 Ü	15	562	15176569	45	
3	SOD 22/16 B	15	577	18908581.4	55	
4	SOD 22/17 Ü	15	577	14996448.5	43	
5	SOD 22/17 IB	15	584	19942758.8	57	
6	divers	15	584	18508220.7	43	
7	divers	15	588	24396793	57	
8	divers	15	584	20265219.3	51	
9	divers	15	584	19279739.6	49	
10	divers	15	582	17156224.8	40	
11	divers	15	579	25437324.1	60	
12	SOD Standard (0.196 mg/ml)	15	571	22398439.5		

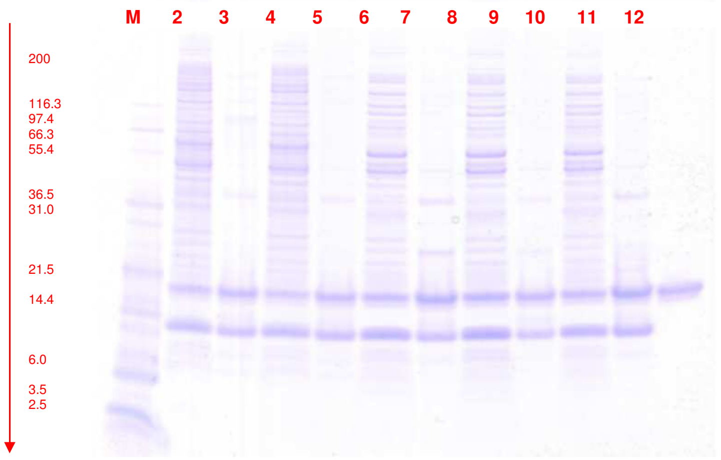

NuPAGE Elektrophorese

Datum: 07.05.2009

Quantifizierung

07052009_SOD24b

Versuch SOD 24 - Verteilung IBs / lösl. Protein

Operator Patricia

Gel Nu PAGE 4-12% Bis Tris Gel
MES running buffer

Marker Mark 12™

Settings 200 V, max. 400 mA, 30´-50´

Scan-Datei 07052009_SOD24b

Bemerkungen

Quantifizierung

well	Probe	red	Vol [µl]	comments
1	MW Standard Mark 12		7	
2	SOD 24/9 Ü	+	15	
3	SOD 24/9 IB	+	15	
4	SOD 24/10 Ü	+	15	
5	SOD 24/10 IB	+	15	
6	SOD 24/11 Ü	+	15	
7	SOD 24/11 IB	+	15	
8	SOD 24/12 Ü	+	15	
9	SOD 24/12 IB	+	15	
10	SOD 24/13 Ü	+	15	
11	SOD 24/13 IB	+	15	
12	SOD Standard (0.196 mg/ml)	+	15	

well	Probe	Vol [µl]	position	raw vol.	ratio [%]	comments
1	MW Standard Mark 12	7				
2	SOD 24/9 Ü	15	682	24812380	94	
3	SOD 24/9 IB	15	682	1712244.45	6	
4	SOD 24/10 Ü	15	682	25195907.1	90	
5	SOD 24/10 IB	15	679	2729897.22	10	
6	SOD 24/11 Ü	15	680	17238934.5	63	
7	SOD 24/11 IB	15	672	9977763.83	37	
8	SOD 24/12 Ü	15	676	27994557	60	
9	SOD 24/12 IB	15	680	18729053.5	40	
10	SOD 24/13 Ü	15	674	26764847.1	56	
11	SOD 24/13 IB	15	680	21062283.4	44	
12	SOD Standard (0.196 mg/ml)	15	672	19245566.8		

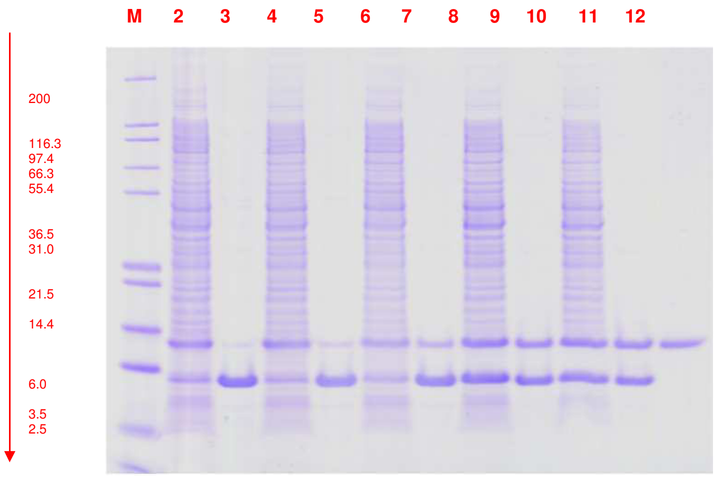

NuPAGE Elektrophorese

Datum: 07.05.2009

Quantifizierung

07052009_SOD24b

Versuch SOD 24 - Verteilung IBs / lösl. Protein

Operator Patricia

Gel Nu PAGE 4-12% Bis Tris Gel
MES running buffer

Marker Mark 12™

Settings 200 V, max. 400 mA, 30´-50´

Scan-Datei 07052009_SOD24b

Bemerkungen

Quantifizierung

well	Probe	red	Vol [µl]	comments
1	MW Standard Mark 12		7	
2	SOD 24/14 IB	+	15	
3	SOD 24/14 U	+	15	
4	SOD 24/15 Ü	+	15	
5	SOD 24/15 IB	+	15	
6	SOD 24/16 Ü	+	15	
7	SOD 24/16 IB	+	15	
8	SOD 24/17 Ü	+	15	
9	SOD 24/17 IB	+	15	
10	divers	+	15	
11	divers	+	15	
12	SOD Standard (0.196 mg/ml)	+	15	

well	Probe	Vol [µl]	position	raw vol.	ratio [%]	comments
1	MW Standard Mark 12	7	0	0		
2	SOD 24/14 IB	15	743	51813862	51	
3	SOD 24/14 U	15	747	50761061	49	
4	SOD 24/15 Ü	15	745	44794540	47	
5	SOD 24/15 IB	15	745	50710329	53	
6	SOD 24/16 Ü	15	747	44282725	47	
7	SOD 24/16 IB	15	745	50896182	53	
8	SOD 24/17 Ü	15	750	43266778	46	
9	SOD 24/17 IB	15	745	49859962	54	
10	divers	15	745	46322993	47	
11	divers	15	745	52632133	53	
12	SOD Standard (0.196 mg/ml)	15	738	51669892		

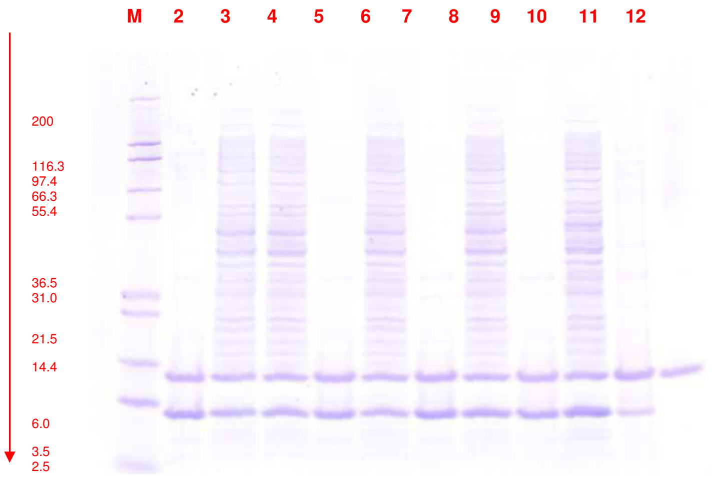

ok

NuPAGE Elektrophorese

Datum: 19.05.2009

Quantifizierung

19052009_SOD25b

Versuch SOD 25 - Verteilung IBs / lösl. Protein

Operator Patricia

Gel Nu PAGE 4-12% Bis Tris Gel
MES running buffer

Marker Mark 12™

Settings 200 V, max. 400 mA, 30´-50´

Scan-Datei 19052009_SOD25b

Bemerkungen

Quantifizierung

well	Probe	red	Vol [µl]	comments
1	MW Standard Mark 12		7	
2	SOD 25/9 Ü	+	15	
3	SOD 25/9 IB	+	15	
4	SOD 25/10 Ü	+	15	
5	SOD 25/10 IB	+	15	
6	SOD 25/11 Ü	+	15	
7	SOD 25/11 IB	+	15	
8	SOD 25/12 Ü	+	15	
9	SOD 25/12 IB	+	15	
10	SOD 25/13 Ü	+	15	
11	SOD 25/13 IB	+	15	
12	SOD Standard (0.196 mg/ml)	+	15	

well	Probe	Vol [µl]	position	raw vol.	ratio [%]	comments
1	MW Standard Mark 12	7				
2	SOD 25/9 Ü	15	592	12174789.3	99	
3	SOD 25/9 IB	15	603	163341.6	1	
4	SOD 25/10 Ü	15	606	14893517.8	91	
5	SOD 25/10 IB	15	606	1500001.27	9	
6	SOD 25/11 Ü	15	606	15608891.2	65	
7	SOD 25/11 IB	15	606	8355365.04	35	
8	SOD 25/12 Ü	15	610	18283119.7	58	
9	SOD 25/12 IB	15	610	13512220.3	42	
10	SOD 25/13 Ü	15	610	14744182.6		
11	SOD 25/13 IB	15	680	21062283.4	59	
12	SOD Standard (0.196 mg/ml)	15	672	19245566.8		

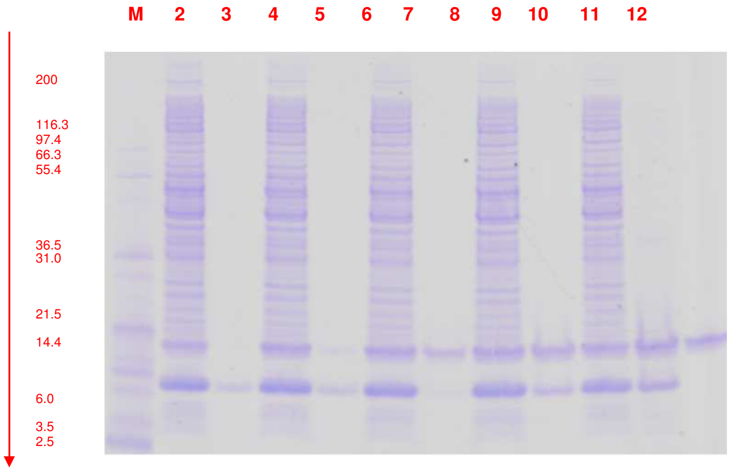

NuPAGE Elektrophorese

Datum: 19.05.2009

Quantifizierung

19052009_SOD25b

Versuch SOD 25 - Verteilung IBs / lösl. Protein

Operator Patricia

Gel Nu PAGE 4-12% Bis Tris Gel
MES running buffer

Marker Mark 12™

Settings 200 V, max. 400 mA, 30´-50´

Scan-Datei 19052009_SOD25b

Bemerkungen

Quantifizierung

well	Probe	red	Vol [µl]	comments
1	MW Standard Mark 12		7	
2	SOD 25/14 Ü	+	15	
3	SOD 25/14 IB	+	15	
4	SOD 25/15 Ü	+	15	
5	SOD 25/15 IB	+	15	
6	SOD 25/16 Ü	+	15	
7	SOD 25/16 IB	+	15	
8	SOD 25/17 Ü	+	15	
9	SOD 25/17 IB	+	15	
10	divers	+	15	
11	divers	+	15	
12	SOD Standard (0.196 mg/ml)	+	15	

well	Probe	Vol [µl]	position	raw vol.	ratio [%]	comments
1	MW Standard Mark 12	7				
2	SOD 25/14 Ü	15	550	160203	53	
3	SOD 25/14 IB	15	552	144146	47	
4	SOD 25/15 Ü	15	552	132671	48	
5	SOD 25/15 IB	15	561	142073	52	
6	SOD 25/16 Ü	15	555	142060	48	
7	SOD 25/16 IB	15	564	152383	52	
8	SOD 25/17 Ü	15	562	152722	47	
9	SOD 25/17 IB	15	562	171135	53	
10	divers	15	561	155967	49	
11	divers	15	566	164983	51	
12	SOD Standard (0.196 mg/ml)	15	559	151352		

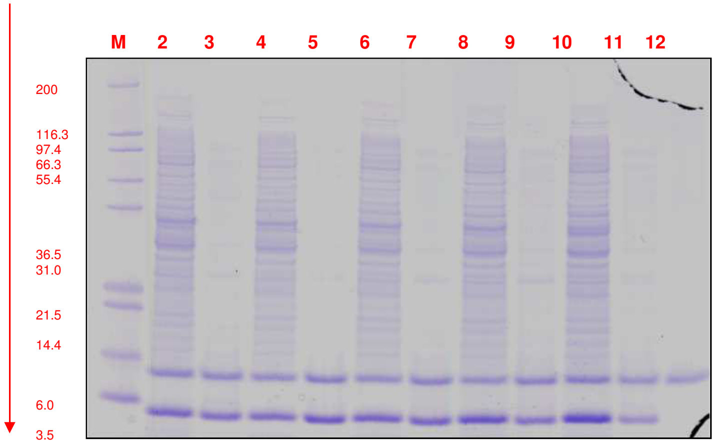

ok
